# Levels of domain-specific physical activity at work, in the household, for travel and for leisure among 327 789 adults from 104 countries

**DOI:** 10.1136/bjsports-2020-102601

**Published:** 2020-11-23

**Authors:** Tessa Strain, Katrien Wijndaele, Leandro Garcia, Melanie Cowan, Regina Guthold, Soren Brage, Fiona C Bull

**Affiliations:** 1 MRC Epidemiology Unit, University of Cambridge, Cambridge, UK; 2 Physical Activity for Health Research Centre, University of Edinburgh, Edinburgh, UK; 3 Centre for Public Health, Queen's University Belfast, Belfast, UK; 4 Department of Noncommunicable Diseases, World Health Organization, Geneva, Switzerland; 5 Maternal, Newborn, Child, and Adolescent Health and Ageing Department, World Health Organization, Geneva, Switzerland; 6 Department of Health Promotion, Division of Universal Health Coverage and Healthier Populations, World Health Organization, Geneva, Switzerland; 7 Physical Activity Unit, The University of Western Australia, Perth, Western Australia, Australia

**Keywords:** physical activity, surveillance, epidemiology

## Abstract

**Objective:**

To compare the country-level absolute and relative contributions of physical activity at work and in the household, for travel, and during leisure-time to total moderate-to-vigorous physical activity (MVPA).

**Methods:**

We used data collected between 2002 and 2019 from 327 789 participants across 104 countries and territories (n=24 low, n=34 lower-middle, n=30 upper-middle, n=16 high-income) from all six World Health Organization (WHO) regions. We calculated mean min/week of work/household, travel and leisure MVPA and compared their relative contributions to total MVPA using Global Physical Activity Questionnaire data. We compared patterns by country, sex and age group (25–44 and 45–64 years).

**Results:**

Mean MVPA in work/household, travel and leisure domains across the 104 countries was 950 (IQR 618–1198), 327 (190–405) and 104 (51–131) min/week, respectively. Corresponding relative contributions to total MVPA were 52% (IQR 44%–63%), 36% (25%–45%) and 12% (4%–15%), respectively. Work/household was the highest contributor in 80 countries; travel in 23; leisure in just one. In both absolute and relative terms, low-income countries tended to show higher work/household (1233 min/week, 57%) and lower leisure MVPA levels (72 min/week, 4%). Travel MVPA duration was higher in low-income countries but there was no obvious pattern in the relative contributions. Women tended to have relatively less work/household and more travel MVPA; age groups were generally similar.

**Conclusion:**

In the largest domain-specific physical activity study to date, we found considerable country-level variation in how MVPA is accumulated. Such information is essential to inform national and global policy and future investments to provide opportunities to be active, accounting for country context.

## Introduction

The physical and mental health benefits of physical activity are well established,^1–3^ yet 27.5% of the global adult population do not reach the minimum recommended levels.^4^ The World Health Organization (WHO) member states agreed to a 15% reduction in physical inactivity levels by 2030^2^ but this is unlikely to be achieved, given current trajectories.^4^

Physical activity is a complex behaviour; however, opportunities to be active exist in several domains in life: at work, household or at school, for travel or during leisure time. Different domains may contribute to health in different ways.^5^ Understanding the composition of how and where people accumulate their activity has important implications for policy, clinical practice and future public health investments. Economic development and its associated technological, demographic and societal changes have the potential to influence the opportunities for activity across different settings.^6 7^ One way of investigating these patterns is by comparing domain-specific physical activity behaviour between countries at different stages of economic development.

Occupational physical activity is influenced by the country’s urbanisation level and occupational structure, with more urbanised, service-dominated countries showing lower levels.^8^ Leisure activity levels appear to be higher in high compared with low-income countries,^9^ although even between high-income countries, levels of urbanisation and wealth seem to be positively associated with leisure activity prevalence.^10^ Active travel patterns are less clear by income group as high-income countries provide examples of both high and low levels. For instance, active travel accounts for half of all journeys in the Netherlands and Switzerland but only a quarter in the UK and California,^11^ suggesting differences in available transport infrastructure, social norms or both. This illustrates the complexity of the global perspective on domain-specific physical activity levels, something that has not been systematically researched.

Older age and female sex are known correlates of lower overall physical activity levels.^12 13^ While a number studies have shown differences in the absolute levels of domain-specific activity,^14–16^ few have explored how the relative contributions by age and sex differ.^17 18^ Understanding whether different at-risk subgroups rely on certain physical activity domains more than others is critical information for policy-makers to protect existing and promote new behaviours and to help reduce health inequalities.

This study uses the latest internationally comparable physical activity data to compare the absolute and relative contributions of physical activity at work and in the household, for travel and during leisure time to total moderate-to-vigorous physical activity (MVPA) for 104 countries across the spectrum of economic development. As a secondary aim, we also compared the domain-specific MVPA contributions by sex and age group.

## Methods

### Data sources

We obtained the most recent individual-level survey data from 104 countries and territories (hereafter called countries) in which the WHO Global Physical Activity Questionnaire (GPAQ) was used.^19^ These included 94 surveys using the WHO STEPwise approach to non-communicable disease surveillance (STEPS),^20^ 6 from the WHO Study on global AGEing and adult health (SAGE),^21^ and 4 from public archives (see Acknowledgments and Data Availability statements for details).

The countries covered were from all six WHO regions: Africa (n=37), Americas (n=15), Eastern Mediterranean (n=12), Europe (n=8), South-East Asia (n=8) and Western Pacific (n=24). According to World Bank Income Classification (2020),^22^ 24 were low, 24 were lower-middle, 30 were upper-middle, and 16 were high-income countries (online supplemental file 1). These countries make up 71% of the global population in 2020.^23^

10.1136/bjsports-2020-102601.supp1 Supplementary data

In addition, an earlier STEPS survey was identified for four countries in the African region (Benin, Botswana, Malawi, Seychelles) for an exploratory analysis of domain-specific trends. These were countries within the same region, spanning all income classification groups, and with national data from two points in time with a minimum time gap of 5 years between them and the most recent time point within the last 5 years.

### Representativeness of data

Survey sampling was designed to obtain a nationally representative (n=90 + 4 trend datasets) or a subnational (n=14) sample. In the majority of cases, this was a stratified multistage clustered design but some of the subnational surveys or smaller islands used simple random, quota or interval sampling, or a census (online supplemental file 2). Sample weights were provided for 91 (+3 trend) surveys to adjust for unequal selection probabilities. These also adjusted for the oversampling of those ≥50 years in the SAGE surveys, and normalised to population distributions of age, sex and in some cases region and ethnicity in the publicly available surveys.

10.1136/bjsports-2020-102601.supp2 Supplementary data

Eight of the subnational surveys were urban only. For these, we imputed rural data and weighted them by national urban–rural prevalence to derive national estimates. In the urban–rural mixed subnational survey, we performed this weighting step with the data collected. The remaining five subnational surveys were analysed with no adjustment. Full methods are provided in online supplemental file 3.

10.1136/bjsports-2020-102601.supp3 Supplementary data

The analysis was limited to the common age-range across all surveys (25–64 years) for comparability reasons so the estimates presented in this paper are not official national statistics.

### Global Physical Activity Questionnaire

We only included surveys which had used the WHO GPAQ. This instrument captures MVPA undertaken in a typical week at work (paid or unpaid, including household chores), for travel to and from places by walking or cycling, and during leisure time (including sports and fitness-enhancing activities).^19^ Work/household and leisure domains are split into moderate and vigorous intensity. Only MVPA undertaken in bouts of 10 min or more is reported, and participants are reminded not to report activities already included in an earlier answer. The GPAQ was translated into national languages, and back translated for quality assurance, and show-cards were used to provide culturally relevant examples of physical activities in each domain.

The GPAQ has shown comparable levels of validity to other self-report instruments across a variety of cultural settings.^24–28^ In the absence of domain-specific validity evidence, we estimated the Pearson’s correlation coefficients for the associations between the country-level domain-specific MVPA (median min/week) and indicators selected *a priori* (online supplemental file 4). These were work/household and the proportion of the workforce in the agricultural sector (males: r=0.43, females: r=0.46) and other occupations involving a high degree of manual labour (males: r=0.47, females: r=0.48); travel and the number of vehicle registrations per adult in the population (r=−0.49); and leisure and the GINI measure of economic inequality across the population (r=0.03), and the Human Development Index (r=0.27).

10.1136/bjsports-2020-102601.supp4 Supplementary data

### Data processing

The standard GPAQ data processing protocol was followed.^19^ Individuals reporting implausible (>16 hours/day in one of the domains) or inconsistent (eg, no days but valid duration) were excluded. We considered moderate and vigorous intensity work/household and leisure physical activity separately for this step. We further excluded those with missing data in either the frequency or duration variables in any domain (online supplemental file 5). We calculated total weekly duration in minutes for each individual by multiplying the number of days and daily time variables for each domain and summing the totals. For the main analysis, we combined the moderate and vigorous components of work/household and leisure as reported. In a sensitivity analysis, we doubled the reported duration of vigorous intensity activity to reflect the physical activity guidelines that suggest 75 min of vigorous intensity activity provides comparable benefits to 150 min of moderate intensity activity.^3^

10.1136/bjsports-2020-102601.supp5 Supplementary data

We estimated the absolute levels of domain-specific MVPA (min/week) at a country level, and by sex and age group (25–44 and 45–64 years) strata based on all those meeting the inclusion criteria. We calculated relative contributions at an individual level, and group-level arithmetic means were presented. Those who reported no MVPA across all domains could not provide any information to the relative contributions (online supplemental file 5). We described differences with reference to the World Bank Income Classification 2020.^22^ We estimated Pearson’s correlation coefficients for the associations between the mean domain-specific MVPA and total MVPA at a country level. We performed the analyses in Stata V.16 (StataCorp) and produced the ternary plots in R Studio using the ggtern^29^ and tricolore packages.^30^

### Public involvement

The experiences of data collectors informed the development of the GPAQ,^24^ and assisted in the cultural and language adaptations made for each country.

## Results

We included data from 327 789 individuals aged 25–64 years from 104 countries collected between 2002 and 2019. The prevalence of meeting the WHO physical activity guidelines ranged between 36% and 98%, and the proportion reporting zero min/week of total MVPA ranged between 1% and 51% (table 1).

**Table 1:** Descriptive characteristics of the survey samples of participants aged 25–64 years from 104 countries

Country	Year	Sample size	N (%) female	N (%) 25–44 years	N (%) reporting 0 min/week overall activity	N (%) meeting the WHO physical activity guidelines
*Low-income countries*						
Afghanistan	2018	2673	1351 (50.6)	1910 (71.5)	561 (21.0)	1865 (69.8)
Benin	2015	3963	1998 (50.4)	2824 (71.3)	454 (11.5)	3308 (83.5)
Burkina Faso	2013	3782	2015 (53.3)	2645 (69.9)	341 (9.0)	3153 (83.4)
Central African Republic	2017	2800	1380 (49.3)	2016 (72.0)	97 (3.5)	2665 (95.2)
Chad	2008	1622	795 (49.0)	1063 (65.5)	245 (15.1)	1196 (73.7)
Democratic Republic of the Congo	2005	1194	740 (62.0)	846 (70.9)	205 (17.2)	902 (75.5)
Eritrea	2010	5380	4579 (85.1)	3792 (70.5)	394 (7.3)	4755 (88.4)
Ethiopia	2006	3879	2257 (58.2)	2219 (57.2)	120 (3.1)	3199 (82.5)
Gambia	2010	3888	2019 (51.9)	2879 (74.1)	599 (15.4)	3172 (81.6)
Guinea	2009	1661	837 (50.4)	1149 (69.2)	101 (6.1)	1457 (87.7)
Liberia	2011	2287	1150 (50.3)	1563 (68.3)	423 (18.5)	1686 (73.7)
Madagascar	2005	3999	2054 (51.4)	2763 (69.1)	374 (9.4)	3369 (84.2)
Malawi	2017	2552	1315 (51.5)	1904 (74.6)	15 (0.6)	2508 (98.3)
Mali	2013	691	457 (66.1)	378 (54.7)	155 (22.4)	449 (65.0)
Mozambique	2005	2853	1618 (56.7)	1982 (69.5)	31 (1.1)	2747 (96.3)
Nepal	2019	4356	2325 (53.4)	2915 (66.9)	202 (4.6)	4069 (93.4)
Niger	2007	2050	921 (44.9)	1180 (57.6)	349 (17.0)	1550 (75.6)
Rwanda	2012–2013	5495	2933 (53.4)	3939 (71.7)	374 (6.8)	4787 (87.1)
Sierra Leone	2009	2413	1248 (51.7)	1692 (70.1)	185 (7.7)	2120 (87.9)
Tajikistan	2016–2017	2108	947 (44.9)	1643 (77.9)	357 (17.0)	1498 (71.1)
Togo	2010–2011	2871	1531 (53.3)	2059 (71.7)	116 (4.0)	2631 (91.6)
Uganda	2014	2820	1449 (51.4)	2099 (74.4)	72 (2.6)	2698 (95.7)
United Republic of Tanzania	2012	8100	4128 (51.0)	5905 (72.9)	203 (2.5)	7693 (95.0)
United Republic of Tanzania (Zanzibar)	2011	2621	1374 (52.4)	1909 (72.8)	92 (3.5)	2308 (88.1)
*Lower-middle-income countries*						
Bangladesh	2018	6866	3456 (50.3)	4497 (65.5)	459 (6.7)	6048 (88.1)
Bhutan	2014	2339	1008 (43.1)	1694 (72.4)	82 (3.5)	2201 (94.1)
Cabo Verde	2007	1724	869 (50.4)	1317 (76.4)	120 (6.9)	1434 (83.2)
Cambodia	2010	5432	2789 (51.3)	3564 (65.6)	298 (5.5)	4960 (91.3)
Cameroon	2003	5253	3289 (62.6)	3618 (68.9)	627 (11.9)	3388 (64.5)
Comoros	2011	4646	2329 (50.1)	3147 (67.7)	252 (5.4)	4082 (87.9)
Côte d'Ivoire	2005	2593	1382 (53.3)	1938 (74.7)	334 (12.9)	1773 (68.4)
Egypt	2017	4984	2478 (49.7)	3235 (64.9)	712 (14.3)	3679 (73.8)
Eswatini	2014	2017	1138 (56.4)	1426 (70.7)	190 (9.4)	1676 (83.1)
Ghana	2007–2008	2985	1463 (49.0)	1800 (60.3)	288 (9.7)	2580 (86.4)
India	2007–2008	7770	3667 (47.2)	4888 (62.9)	449 (5.8)	7026 (90.4)
Kenya	2015	3421	1732 (50.6)	2529 (73.9)	103 (3.0)	3234 (94.5)
Kiribati	2015–2016	1618	866 (53.5)	1032 (63.8)	332 (20.5)	1043 (64.4)
Kyrgyzstan	2013	2620	1297 (49.5)	1639 (62.6)	178 (6.8)	2320 (88.6)
Lao People’s Democratic Republic	2013	2124	1234 (58.1)	1244 (58.5)	58 (2.7)	1946 (91.6)
Lesotho	2012	1791	924 (51.6)	1362 (76.0)	33 (1.8)	1710 (95.5)
Mauritania	2006	1095	609 (55.6)	587 (53.6)	190 (17.4)	571 (52.1)
Micronesia, Fed. Sts.	2016	1643	823 (50.1)	1142 (69.5)	529 (32.2)	1000 (60.9)
Moldova, Republic of	2013	3805	1848 (48.6)	2156 (56.7)	218 (5.7)	3409 (89.6)
Mongolia	2019	5429	2768 (51.0)	3506 (64.6)	853 (15.7)	4089 (75.3)
Morocco	2017	4088	2106 (51.5)	2511 (61.4)	490 (12.0)	3296 (80.6)
Myanmar	2014	8143	4052 (49.8)	4959 (60.9)	701 (8.6)	6872 (84.4)
Niue	2011–2012	581	319 (54.9)	271 (46.6)	10 (1.7)	554 (95.4)
Pakistan	2013–2014	5309	3101 (58.4)	3644 (68.6)	1426 (26.9)	3156 (59.5)
Papua New Guinea	2007–2008	1899	913 (48.1)	1368 (72.0)	132 (7.0)	1670 (88.0)
Solomon Islands	2015	1912	999 (52.3)	1328 (69.4)	197 (10.3)	1592 (83.2)
Sudan	2016	5799	2742 (47.3)	4033 (69.5)	325 (5.6)	4952 (85.4)
São Tomé and Principe	2008	2261	1192 (52.7)	1558 (68.9)	147 (6.5)	1975 (87.4)
Timor-Leste	2014	1792	723 (40.3)	1196 (66.7)	130 (7.3)	1455 (81.2)
Tokelau	2014	439	240 (54.6)	261 (59.5)	28 (6.3)	386 (87.9)
Vanuatu	2011	4457	2343 (52.6)	3031 (68.0)	91 (2.0)	4177 (93.7)
Viet Nam	2015	3116	1605 (51.5)	1866 (59.9)	556 (17.9)	2302 (73.9)
West Bank and Gaza Strip	2010–2011	5105	2536 (49.7)	3580 (70.1)	1336 (26.2)	3050 (59.7)
Zambia	2017	2550	1253 (49.1)	1916 (75.1)	139 (5.5)	2276 (89.3)
*Upper-middle-income countries*						
Algeria	2016–2017	5566	2789 (50.1)	3677 (66.1)	741 (13.3)	4193 (75.3)
American Samoa	2004	2015	1010 (50.1)	1354 (67.2)	941 (46.7)	845 (41.9)
Armenia	2016	1825	877 (48.1)	1046 (57.3)	283 (15.5)	1437 (78.8)
Azerbaijan	2017	2322	1196 (51.5)	1343 (57.8)	269 (11.6)	1889 (81.3)
Belarus	2016–2017	4203	2178 (51.8)	2162 (51.4)	301 (7.2)	3682 (87.6)
Botswana	2014	2688	1343 (49.9)	1939 (72.2)	270 (10.0)	2118 (78.8)
Brazil	2013–2014	21 942	11 692 (53.3)	12 721 (58.0)	6105 (27.8)	14 509 (66.1)
China	2008–2010	9055	4574 (50.5)	4235 (46.8)	1187 (13.1)	7130 (78.7)
Cook Islands	2013–2015	1064	536 (50.4)	636 (59.8)	169 (15.8)	800 (75.2)
Ecuador	2018	3620	1879 (51.9)	2041 (56.4)	304 (8.4)	2977 (82.2)
Fiji	2011	2325	1173 (50.4)	1369 (58.9)	144 (6.2)	1973 (84.9)
Gabon	2009	1958	1144 (58.4)	1269 (64.8)	231 (11.8)	1418 (72.4)
Georgia	2016	3346	1743 (52.1)	1726 (51.6)	322 (9.6)	2776 (83.0)
Grenada	2010–2011	1030	502 (48.8)	674 (65.4)	184 (17.8)	733 (71.1)
Guyana	2016	2051	1017 (49.6)	1254 (61.1)	391 (19.1)	1444 (70.4)
Iraq	2015	3036	1505 (49.6)	1981 (65.2)	853 (28.1)	1615 (53.2)
Jordan	2019	4350	2325 (53.5)	2979 (68.5)	476 (10.9)	3250 (74.7)
Lebanon	2017	1426	764 (53.6)	885 (62.1)	726 (50.9)	548 (38.4)
Libya	2009	3460	1740 (50.3)	2692 (77.8)	783 (22.6)	2194 (63.4)
Maldives	2011	1222	615 (50.3)	911 (74.6)	320 (26.2)	756 (61.9)
Marshall Islands	2002	1806	1100 (60.9)	1271 (70.4)	751 (41.6)	997 (55.2)
Mexico	2009–2010	1324	717 (54.1)	906 (68.4)	192 (14.5)	962 (72.6)
Nauru	2015–2016	1031	534 (51.8)	719 (69.8)	267 (25.9)	617 (59.8)
Russian Federation	2007–2010	2306	1284 (55.7)	1248 (54.1)	115 (5.0)	2146 (93.1)
Saint Lucia	2012	1673	1039 (62.1)	853 (51.0)	268 (16.0)	1226 (73.3)
Samoa	2013	1412	681 (48.2)	901 (63.8)	118 (8.4)	1216 (86.1)
South Africa	2007–2008	2447	1291 (52.8)	1476 (60.3)	915 (37.4)	1374 (56.2)
Sri Lanka	2014–2015	4321	2184 (50.6)	2436 (56.4)	837 (19.4)	3029 (70.1)
Tonga	2017	3201	2068 (64.6)	1940 (60.6)	826 (25.8)	1920 (60.0)
Tuvalu	2015	935	489 (52.3)	590 (63.1)	141 (15.1)	689 (73.6)
*High-income countries*						
Anguilla	2016	1347	695 (51.6)	782 (58.0)	235 (17.4)	1014 (75.3)
Bahamas	2011–2012	1617	806 (49.8)	998 (61.7)	524 (32.4)	955 (59.0)
Barbados	2007	934	489 (52.4)	566 (60.6)	207 (22.2)	599 (64.1)
British Virgin Islands	2009	1065	486 (45.6)	572 (53.7)	189 (17.8)	780 (73.3)
Brunei Darussalam	2015–2016	2761	1418 (51.4)	1793 (64.9)	318 (11.5)	2050 (74.2)
Cayman Islands	2012	1266	624 (49.3)	800 (63.2)	222 (17.5)	922 (72.8)
Chile	2016–2017	3831	1919 (50.1)	2108 (55.0)	668 (17.4)	2699 (70.4)
French Polynesia	2010	2862	1379 (48.2)	1815 (63.4)	225 (7.9)	2440 (85.3)
Korea, Rep.	2018	4038	1987 (49.2)	1938 (48.0)	1274 (31.6)	1866 (46.2)
Kuwait	2014	3160	1678 (53.1)	2170 (68.7)	1613 (51.0)	1131 (35.8)
Palau	2016	1173	553 (47.1)	569 (48.5)	261 (22.3)	783 (66.8)
Qatar	2012	2003	1006 (50.2)	1411 (70.5)	466 (23.3)	1182 (59.0)
Seychelles	2013–2014	1239	618 (49.8)	716 (57.8)	59 (4.7)	1007 (81.3)
Trinidad and Tobago	2011	2151	1113 (51.7)	1287 (59.8)	613 (28.5)	1285 (59.8)
USA	2017–2018	3628	1855 (51.1)	1800 (49.6)	726 (20.0)	2504 (69.0)
Uruguay	2013–2014	2090	1091(52.2)	1157 (55.4)	302 (14.5)	1613 (77.2)

Cameroon, Central African Republic, Chad, Côte d’Ivoire, Democratic Republic of the Congo, Ethiopia, Gabon, Guinea, Madagascar, Maldives, Mali, Mauritania, Micronesia Fed. Sts. and Pakistan are subnational surveys. Total sample size is unweighted, all other N/proportions are weighted when sample weights have been provided. Meeting the WHO physical activity guidelines is defined as ≥150 min of moderate intensity activity per week, or ≥75 min of vigorous intensity activity per week, or ≥600 MET-min/week.

### Absolute levels of domain-specific activity

**Figure 1:**
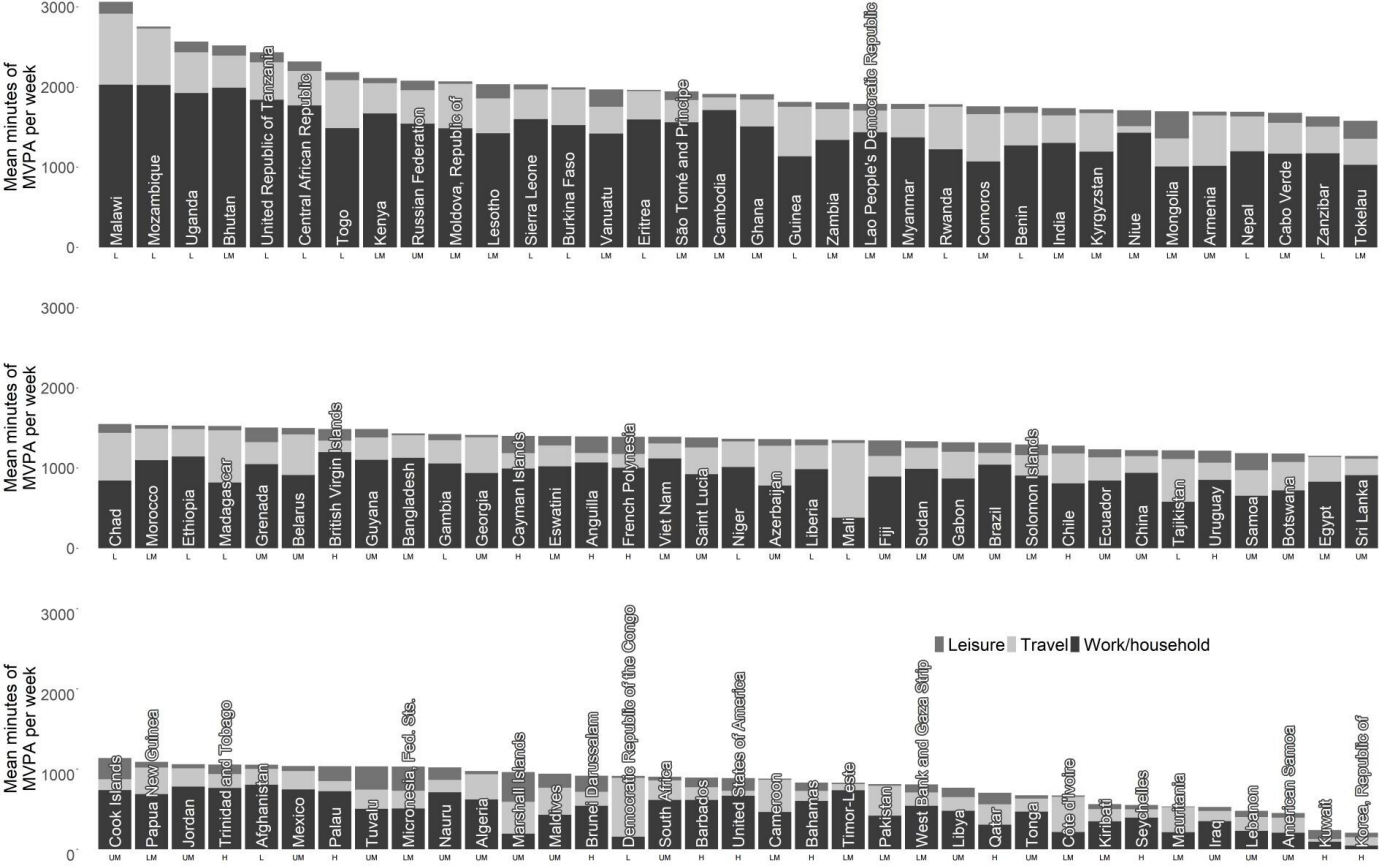
The mean total min/week of moderate-to-vigorous physical activity (MVPA) by domain, across 104 countries, ordered by total MVPA. H=high-income, LM=lower-middle-income, UM=upper-middle-income, L=low-income according to the World Bank Classification 2020. Cameroon, Central African Republic, Chad, Côte d’Ivoire, Democratic Republic of the Congo, Ethiopia, Gabon, Guinea, Madagascar, Maldives, Mali, Mauritania, Micronesia Fed. Sts. and Pakistan are subnational surveys. See online supplemental file 6 for the mean and median domain-specific min/week.

The distributions of the reported weekly durations of work/household, travel and leisure MVPA were highly skewed for all countries; the mode was almost always zero min/week (online supplemental file 6). The country-level means for the domains were 950 (IQR 618–1198), 327 (190–405) and 104 (51–131) min/week, for work/household, travel and leisure time, respectively (table 2, figure 1, online supplementary file 6). Mean work/household MVPA in low-income countries was almost twice that of high-income countries (1233 vs 668 min/week). However, the reverse was seen for mean leisure MVPA (72 vs 143 min/week). Travel MVPA was approximately three times higher in low-income versus high-income countries (499 vs 158 min/week). We found a very strong correlation between work/household MVPA and total MVPA (r=0.95) at the country level. The correlation was 0.58 for travel and 0.04 for leisure MVPA.

10.1136/bjsports-2020-102601.supp6 Supplementary data

**Table 2:** Mean domain-specific min/week, relative contributions and rank order for the 104 countries, by income classification, sex and age group

	All countries	World Bank income classification				Men	Women	25–44 years	45–64 years
		Low	Lower-middle	Upper-middle	High				
Number of countries	104	24	34	30	16	104	104	104	104
Mean min/week (IQR)									
Work/household	950 (618–1198)	1233 (915–1600)	1069 (735–1424)	740 (505–923)	668 (468–924)	1137 (805–1501)	777 (457–1036)	986 (667–1268)	883 (523–1149)
Travel	327 (190–405)	499 (348–609)	320 (260–386)	287 (188–338)	158 (110–184)	373 (237–470)	284 (147–365)	328 (182–410)	323 (204–418)
Leisure	104 (51–131)	72 (35–109)	97 (43–124)	118 (52–157)	143 (108–193)	141 (71–184)	70 (27–99)	120 (59–158)	76 (30–102)
Mean relative contribution to total MVPA (IQR)									
Work/household	52.3 (44.3–63.3)	57.3 (54.1–68.0)	57.2 (48.8–68.8)	47.5 (41.6–56.7)	43.7 (39.3–54.8)	53.0 (46.0–63.3)	51.6 (42.0–65.4)	52.7 (44.7–64.4)	51.6 (43.7–63.9)
Travel	36.0 (25.3–45.3)	38.3 (28.0–39.4)	34.6 (24.5–42.7)	39.7 (29.0–48.8)	28.5 (19.6–34.1)	33.8 (24.1–42.3)	38.3 (26.3–47.7)	34.4 (24.2–42.2)	39.0 (27.6–48.0)
Leisure	11.7 (4.4–15.4)	4.4 (2.5–5.8)	8.2 (3.7–10.8)	12.8 (8.3–15.7)	27.8 (20.5–33.3)	13.2 (5.9–17.7)	10.1 (2.6–14.2)	12.9 (4.7–18.1)	9.3 (2.9–12.4)
Rank order (n)									
W>T> L	70	20	29	17	4	68	65	71	64
W>L>T	10	0	1	1	8	13	6	12	8
L>T>W	1	0	0	0	1	1	0	0	1
L>W>T	0	0	0	0	0	0	4	2	0
T>L>W	2	0	0	1	1	3	2	3	2
T>W> L	21	4	4	11	2	19	27	16	29

L, leisure; T, travel; W, work/household.

### Relative contributions of domain-specific activity to total MVPA

The 47 946 individuals who reported 0 min/week of total MVPA did not contribute to the relative analyses (denominator of zero; online supplemental file 5). Based on the remaining 279 843, the mean contribution of work/household MVPA to total MVPA was 52% (IQR 44%–63%); the respective means for travel and leisure were 36% (25%–45%) and 12% (4%–15%; table 2). Work/household domain was the largest contributor to total MVPA in 80 of the 104 countries, and 70 of these had travel as the second-largest contributor. Travel was the largest contributor in 23 countries, and leisure was dominant in only one.

**Figure 2:**
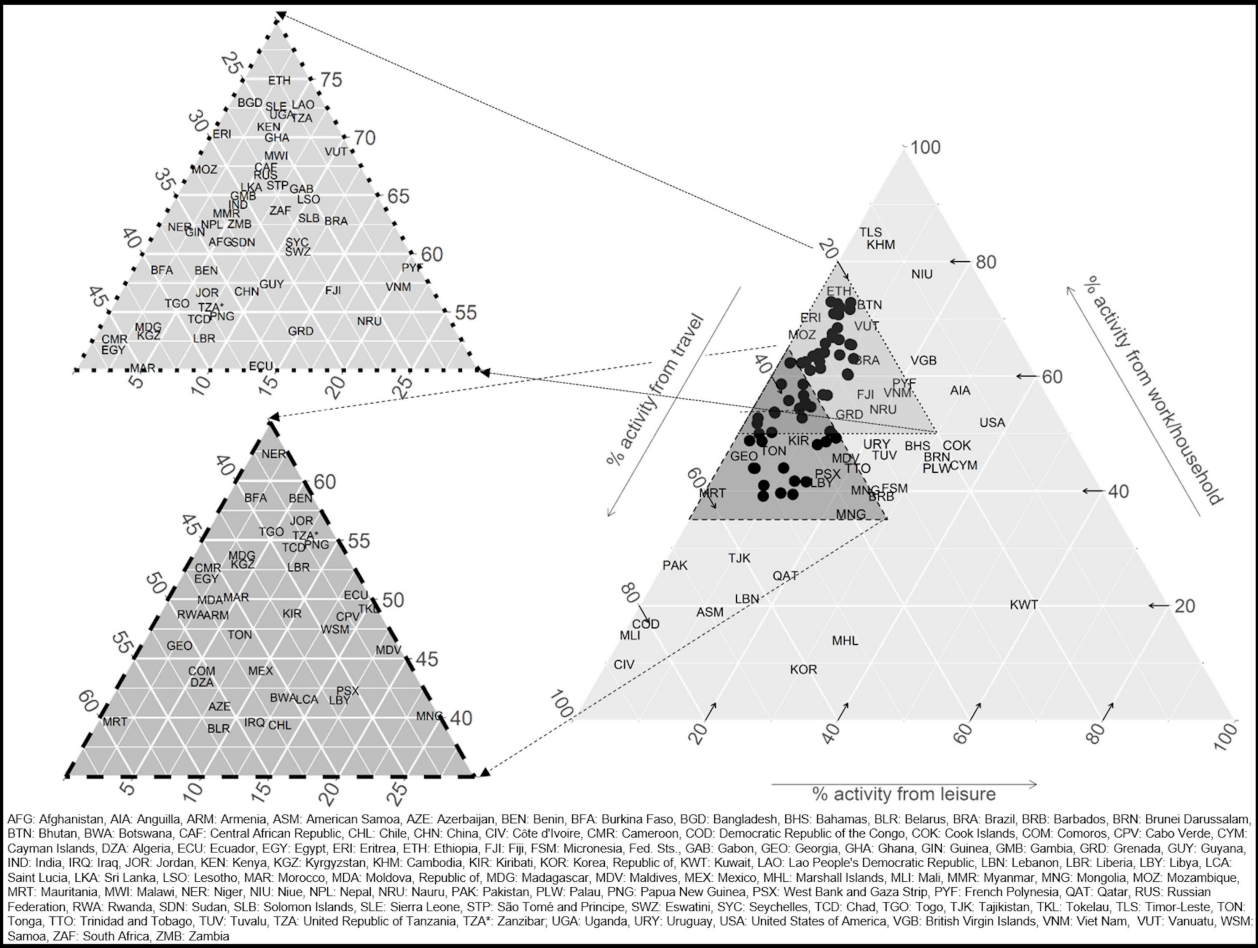
Ternary plot of the relative contributions of work/household, travel and leisure moderate-to-vigorous physical activity (MVPA) to total MVPA. Mean relative contributions should be read following the direction of the arrows for each axis. For example, in the USA, relative contributions are 52% from work/household, 11% from travel, 37% from leisure. Cameroon, Central African Republic, Chad, Côte d’Ivoire, Democratic Republic of the Congo, Ethiopia, Gabon, Guinea, Madagascar, Maldives, Mali, Mauritania, Micronesia Fed. Sts. and Pakistan are subnational surveys.

There was a tendency towards higher contributions of leisure MVPA in high-income countries (mean of 28% compared with 4%, 8%, and 13% in low, lower-middle, and upper-middle-income countries, respectively; table 2, figure 2, online supplementary files 7 and 8). The mean contributions of work/household MVPA in low and lower-middle-income countries were 57%, but were 44%–47% for upper-middle and high-income countries. The contribution of travel to total MVPA did not follow a clear pattern by World Bank income group.

10.1136/bjsports-2020-102601.supp7 Supplementary data

10.1136/bjsports-2020-102601.supp8 Supplementary data

Doubling the duration of vigorous intensity activity (ie, 1 min of vigorous physical activity=2 min of moderate intensity physical activity) in the work/household and leisure domain obviously influenced the absolute durations but did not affect the relative contributions in a meaningful way. This was because those individuals reporting high levels of work/household vigorous intensity activity were already obtaining close to 100% of their MVPA in this domain and so further increases in the absolute durations did not shift the balance of the domain contributions. This conversion therefore had limited influence on the country-level mean relative contributions which varied by a maximum of 2 percentage points for all domains and the highest contributing domain only changed in five countries.

**Figure 3:**
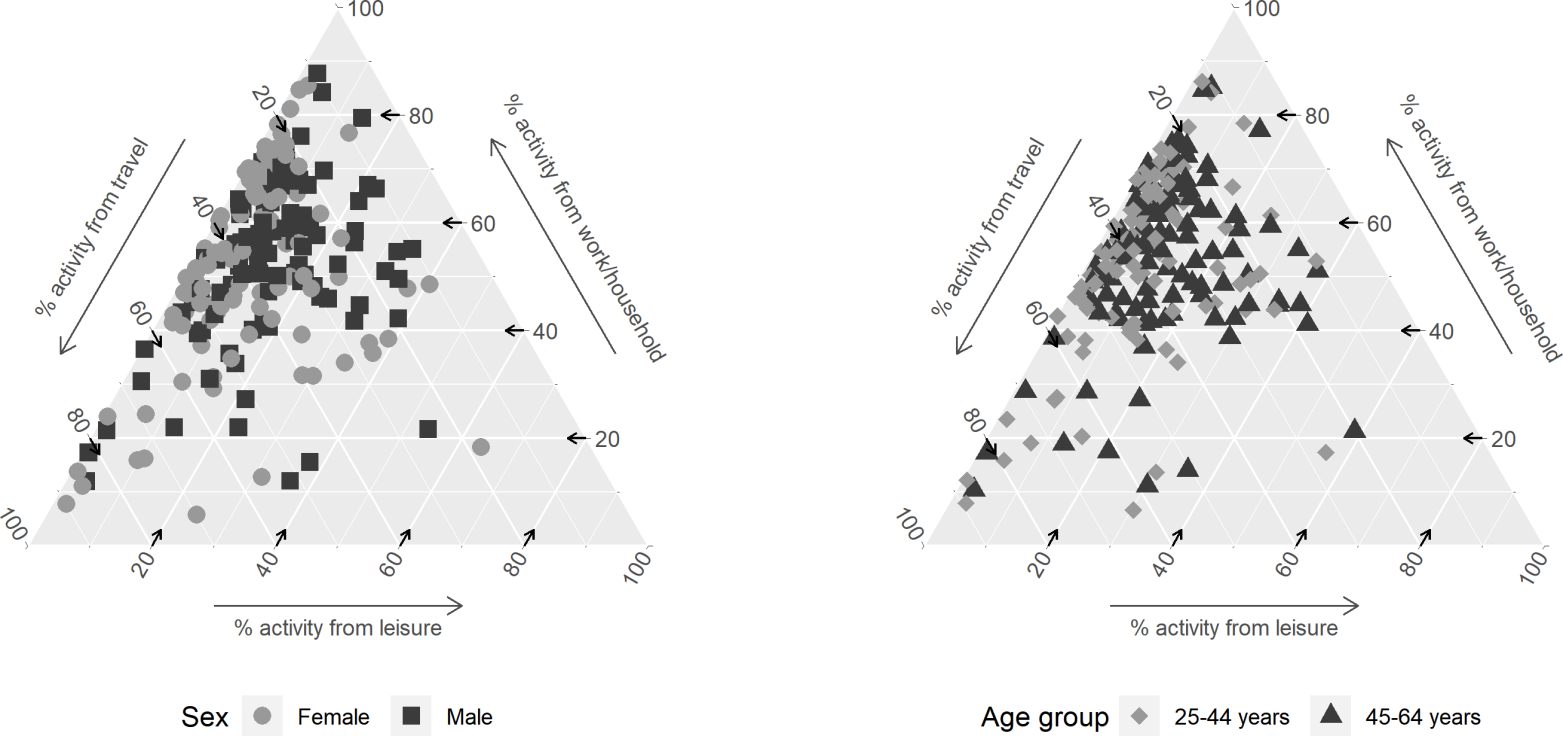
Ternary plots of the relative contributions of work/household, travel and leisure moderate-to-vigorous physical activity (MVPA) to total MVPA by sex and age group. Mean relative contributions should be read following the direction of the arrows for each axis. Cameroon, Central African Republic, Chad, Côte d’Ivoire, Democratic Republic of the Congo, Ethiopia, Gabon, Guinea, Madagascar, Maldives, Mali, Mauritania, Micronesia Fed. Sts. and Pakistan are subnational surveys.

There was a tendency towards greater travel and lower work/household contributions among women compared with men, judged by the means and rank ordering of the domains (table 2, figure 3, online supplementary file 9). Twenty-eight countries had a different ordering of the domain-specific contributions and 42 countries had at least one domain where the relative contributions differed by over 10 percentage points. Differences were more apparent in high-income and upper-middle-income countries.

10.1136/bjsports-2020-102601.supp9 Supplementary data

The variations between 25–44 and 45–64 year olds were not as pronounced as the sex differences. Only 15 countries had over10 percentage point difference in the relative contributions of a domain, and no clear pattern was apparent in terms of the domains in which these occurred (table 2, figure 3, online supplementary file 9). Twelve of these 15 countries were classified as upper-middle and high-income.

### Exploratory analysis of domain-specific trends

**Figure 4:**
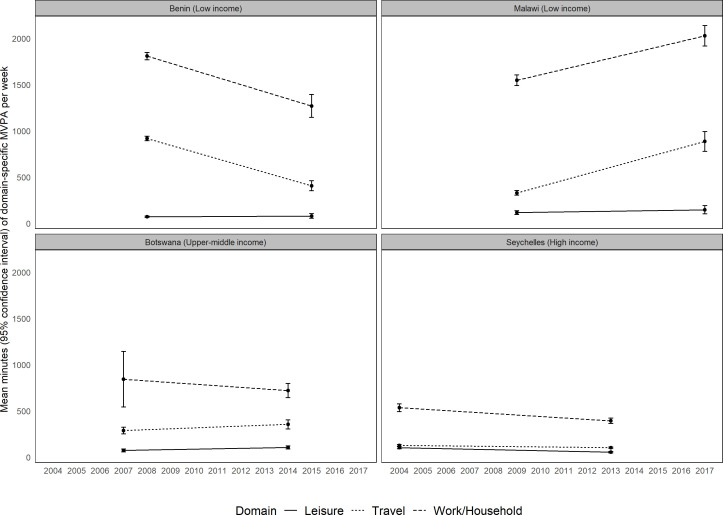
An exploratory analysis of trends in the domain-specific moderate-to-vigorous physical activity (MVPA) in four African countries. World Bank Income 2020 classifications used.

We conducted exploratory analysis of trends using a subsample of data from four African countries (n=25 749), spanning the income classifications (figure 4). The results show considerable heterogeneity in the directions and magnitudes of the changes in domain-specific MVPA over time, even within income classifications.

## Discussion

### Main findings

This study is the largest comparison of country-level domain-specific physical activity to date, including 372 789 individuals from 104 countries. We found the work and household domain to be the highest contributor to total MVPA levels in three-quarters of countries. Travel was the second-largest contributor for all but one of the remaining countries, and was rarely the lowest contributor. There was a trend towards greater contributions of MVPA through work/household in lower income countries, and higher contributions of physical activity through leisure in high-income countries. This pattern was suspected but is shown quantitatively for the first time in a large sample of representative surveys across multiple regions. There were differences in the relative contributions of the domains by sex and age group within countries, with a tendency towards greater differences in upper-middle and high-income countries.

### Comparison with previous results

Previous national-level domain-specific analyses have been focused on specific regions, for example, Africa,^31^ South America,^16^ Asia-Pacific,^14^ while other cross-national studies have investigated the proportions reporting a certain threshold of domain-specific activity (eg, 0, 10, 150 min/week) which limit comparisons with the present results.^15 16 32^ Our results extend the range of comparisons to be cross continental allowing greater comparisons between different World Bank income classifications, and show the relative importance of the domains in addition to the absolute values. Although high-income countries are relatively under-represented in our analyses, the 16 that are included act as helpful comparators to contrast the dominance of work/household-related activity and the low prevalence of leisure activity among many low and middle-income countries. Comparisons in total and domain-specific physical activity by country-level and individual-level income groups is an important yet under-researched area of global physical activity surveillance. However, it is clear that factors beyond income level and economic development, such as cultural and historical aspects, likely play a role in explaining differences between countries.

Our exploratory analysis of changes over time within domains is an indicator of this complexity. Some but not all countries showed a decline in the work/household domain, a feature that was expected as the epidemiological transition is accompanied by a shift away from manual labour and increased access to labour-saving technology in the home.^8^ The variations seen in the work/household and travel domain trajectories is likely to be explained by differences in countries’ socioeconomic circumstances, level of urbanisation and rates of development. Urbanisation and uptake of digital technologies as well as other cultural, environmental and social factors influence the opportunities for increasing levels of participation in the different domains in different ways. These results indicate the importance of further research on this topic with a larger set of comparable data. It is also a reminder that while global comparisons can identify broad patterns, this needs to be coupled with a local understanding of the context in order to develop appropriate policies and interventions.

Building on previous research that has shown lower levels of overall MVPA among women compared with men across the majority of countries worldwide,^4 12^ we showed that the domain composition also varies. The profile for women, at least in high-income and upper-middle-income countries, had a slightly higher contribution of travel and lower contribution of work/household. These results provide a more nuanced understanding of how physical activity varies by sex.

We did not identify any notable differences in the relative contributions of the activity domains between 25–44 and 45–64 year olds, although where differences were found, they tended to be in upper-middle and high-income countries. Research from UK samples indicated that the greater shifts in domain-related contributions occur after 65 years, that is, around the age of retirement.^17 18^

### Implications of the findings

These results can inform the development of interventions and policies aimed at increasing overall physical activity levels as called for by the 2018 Global Action Plan on Physical Activity.^2^ The high contribution of work/household activity for many low and middle-income countries is important for a number of reasons. First, it identifies a reliance on this domain for meeting activity recommendations that may not persist as countries develop economically.^7 8^ Policy action is necessary to ensure that alternative types of activity are available and affordable to those who might otherwise decrease their activity levels as consequence of such societal and economic changes. The travel domain is an obvious area of focus as our results show that it already is an important contributor to overall levels across all countries. Policies and infrastructure development that promote walking and cycling would also support efforts to reduce air pollution and carbon emissions to combat climate change as set out in the Sustainable Development Goals.^33^ Second, these data stress the importance of assessing all domains of physical activity, since solely concentrating on the leisure domain (as has often been the case^34^) would grossly misrepresent, and for many populations underestimate, overall physical activity prevalence.^35^ Third, if domain-specific activity were to show any differential health benefits, as recently debated,^36^ mapping absolute and relative profiles of domain-specific activity is critical for estimations of global disease burden.

### Limitations

Methodological limitations include the unavailability of sampling weights for 13 countries and the necessity of using imputation methods to account for urban–rural variations when including subnational samples. Our aim was to be as inclusive as possible with the available data. Nonetheless, imputation likely increases the error regarding the representativeness of these estimates, over and above that of the national sampling. To maintain comparability between samples, we restricted the age range to 25–64 year olds. For these reasons, the descriptive statistics of overall activity and the proportions meeting the guidelines do not replace any official national statistics and are presented for context only. The novel estimates of the domain-specific relative contributions are valid in the context of this paper, but further work is necessary to generate official statistics that include adults of all ages, and the multitude of other national survey data collected using alternative questionnaires.

The validity of domain-specific GPAQ estimates has not been evaluated against an appropriate criterion, such as a domain log.^37^ Some have raised concerns about the implausible work/household values.^38^ Inaccurate recall, social desirability and activity misclassification are possible sources of error. In lieu of such a validity study, the correlations presented between the work/household and travel domains and the selected country-level indicators provide tentative support for domain-specific analysis using GPAQ data. We believe the weaker results for the leisure domain are in part due to the number of countries with a median MVPA of zero min/week and the lack of specificity in the macro-economic indicators. In addition, the GPAQ states a minimum 10 min bout length, a requirement that is no longer included in the WHO physical activity guidelines.^1^ The inclusion of shorter bouts might alter the duration of reported activity across all domains, but it is difficult to predict which domains would be more affected as the magnitude of these increments is likely context specific. This would influence the relative contributions. Also, the GPAQ does not contain questions about light-intensity activity which is a major contributor to overall activity.^39^ It is likely that its inclusion would change the relative domain-specific results as well the absolute.

Finally, in the relative contribution analysis, we presented the arithmetic mean values (of individuals for each country, and of countries within income classifications). Although the scaled geometric mean is often used in compositional data, this was not an option when so many individuals reported 0 min/week in at least one domain. However, our analysis method is somewhat protected against individuals reporting very high values of domain-specific activity as the relative contributions were calculated at an individual level, before being summarised at a country level. This means a high value in one domain is limited to a maximum of 100% relative contribution. Of course, this does not protect against issues of differential validity between the domains, which is a plausible concern as even the ordering of questions can affect reporting.^40^ In the GPAQ, the work/household domain is asked about first and all surveys used the same ordering. There is also the potential for cultural differences in the reporting as there are different interpretations of work/household, travel and leisure activity across populations. We were also unable to account for seasonal differences as surveys are often conducted within a short period of time that does not span all seasons.

### Future directions

Filling in the data gaps will require wider adoption of the GPAQ in national surveys or harmonisation between different existing questionnaires. This would be pertinent for the European region as many countries have nationally representative data using alternative questionnaires. Nonetheless, it is rare in physical activity studies for the majority of data to be from low and middle-income countries and so this present work does provide an important perspective on the topic.

Future work could extend these methods to investigate subnational regional differences that others have shown to exist.^41 42^ Exploring variation by socioeconomic differences would also have utility, and require suitable harmonisation of indicators of socioeconomic position across nations.

We have also highlighted the need for analysis of trends in domain-specific data. As countries repeat their STEPS surveys, more comparable data will become available. In the meantime, efforts should be made to harmonise existing data.

Lastly, although the present study reflects the optimal analysis of currently available data, the advent of combined location and physical activity sensors may allow objective assessment of domain-specific activity in future research.

### Conclusion

This study is the largest analysis of domain-specific physical activity to date comparing data from 104 countries. Activity at work/household is the main contributor to total MVPA levels, particularly in lower income countries. Leisure activity was the smallest contributor but was highest in high-income countries. Achieving the 15% decrease in global levels of physical inactivity by 2030 as outlined in the Global Action Plan on Physical Activity,^2^ and agreed by 194 Member States of WHO^43^ will require detailed understanding of the context in which people accumulate their physical activity, especially for countries currently undergoing rapid economic development and urbanisation. Across all nations, policy actions will need to align efforts to support and enable opportunities to be active in different domains according to the social, economic and demographic changes, population needs and local context.

**What are the new findings?**

- Physical activity in the domains of work and household, travel and leisure vary broadly across countries in both their absolute levels and relative contributions to total moderate-to-vigorous physical activity (MVPA).
- Work/household MVPA provided the largest contribution in 80 of the 104 countries included in this study.
- Women tended to have relatively less work/household and more travel MVPA.
- Differences between 25–44 and 45–64 year olds were less apparent than differences between the sexes, but were most evident in high and upper-middle-income countries.

**How might it impact on clinical practice in the near future?**

- Clinicians and policy-makers alike should be aware that MVPA is accumulated across a variety of domains, and this varies across countries.
- Work/household is a dominant source of MVPA for many; this has implications for continuing to meet guidelines at the point of changing jobs or retirement.
- Women tended to have relatively lower contributions of work/household and more travel MVPA and this should be considered when tailoring behavioural advice.

## References

[1] Bull FC, Al-Ansari SS, Biddle S, et al World Health Organization 2020 guidelines on physical activity and sedentary behaviour. Brit J Sport Med 2020. doi: 10.1136/bjsports-2020-102955. PMC7719906 33239350

[2] World Health Organization Global Action Plan on Physical Activity 2018–2030: more active people for a healthier world. Geneva: World Health Organization, 2018.

[3] World Health Organization Global recommendations on physical activity for health. Geneva: World Health Organisation, 2010. 26180873

[4] Guthold R, Stevens GA, Riley LM, et al Worldwide trends in insufficient physical activity from 2001 to 2016: a pooled analysis of 358 population-based surveys with 1·9 million participants. Lancet Glob Health 2018;6:e1077–86. 10.1016/S2214-109X(18)30357-7 30193830

[5] Samitz G, Egger M, Zwahlen M Domains of physical activity and all-cause mortality: systematic review and dose-response meta-analysis of cohort studies. Int J Epidemiol 2011;40:1382–400. 10.1093/ije/dyr112 22039197

[6] Ng SW, Norton EC, Popkin BM Why have physical activity levels declined among Chinese adults? findings from the 1991-2006 China health and nutrition surveys. Soc Sci Med 2009;68:1305–14. 10.1016/j.socscimed.2009.01.035 19232811 PMC2731106

[7] Katzmarzyk PT, Mason C The physical activity transition. J Phys Act Health 2009;6:269–80. 10.1123/jpah.6.3.269 19564654

[8] Ng SW, Popkin BM Time use and physical activity: a shift away from movement across the globe. Obes Rev 2012;13:659–80. 10.1111/j.1467-789X.2011.00982.x 22694051 PMC3401184

[9] Lear SA, Hu W, Rangarajan S, et al The effect of physical activity on mortality and cardiovascular disease in 130 000 people from 17 high-income, middle-income, and low-income countries: the PURE study. Lancet 2017;390:2643–54. 10.1016/S0140-6736(17)31634-3 28943267

[10] Van Tuyckom C Macro-environmental factors associated with leisure-time physical activity: a cross-national analysis of EU countries. Scand J Public Health 2011;39:419–26. 10.1177/1403494810396553 21273227

[11] Götschi T, Tainio M, Maizlish N, et al Contrasts in active transport behaviour across four countries: how do they translate into public health benefits? Prev Med 2015;74:42–8. 10.1016/j.ypmed.2015.02.009 25724106 PMC4456468

[12] Mielke GI, da Silva ICM, Kolbe-Alexander TL, et al Shifting the physical inactivity curve worldwide by closing the gender gap. Sports Med 2018;48:481–9. 10.1007/s40279-017-0754-7 28647914

[13] Bauman AE, Reis RS, Sallis JF, et al Correlates of physical activity: why are some people physically active and others not? Lancet 2012;380:258–71. 10.1016/S0140-6736(12)60735-1 22818938

[14] Bauman A, Ma G, Cuevas F, et al Cross-national comparisons of socioeconomic differences in the prevalence of leisure-time and occupational physical activity, and active commuting in six Asia-Pacific countries. J Epidemiol Community Health 2011;65:35–43. 10.1136/jech.2008.086710 20943821

[15] Mitáš J, Cerin E, Reis RS, et al Do associations of sex, age and education with transport and leisure-time physical activity differ across 17 cities in 12 countries? Int J Behav Nutr Phys Act 2019;16:121. 10.1186/s12966-019-0894-2 31796070 PMC6888920

[16] Werneck AO, Baldew S-S, Miranda JJ, et al Physical activity and sedentary behavior patterns and sociodemographic correlates in 116,982 adults from six South American countries: the South American physical activity and sedentary behavior network (SAPASEN). Int J Behav Nutr Phys Act 2019;16:68. 10.1186/s12966-019-0839-9 31429772 PMC6701122

[17] Strain T, Fitzsimons C, Foster C, et al Age-related comparisons by sex in the domains of aerobic physical activity for adults in Scotland. Prev Med Rep 2016;3:90–7. 10.1016/j.pmedr.2015.12.013 26844194 PMC4733093

[18] Bélanger M, Townsend N, Foster C Age-related differences in physical activity profiles of English adults. Prev Med 2011;52:247–9. 10.1016/j.ypmed.2011.02.008 21338622

[19] World Health Organization Global physical activity surveillance. Available: https://www.who.int/ncds/surveillance/steps/GPAQ/en/

[20] World Health Organization STEPwise approach to noncommunicable disease risk factor surveillance (STEPS). Available: https://www.who.int/ncds/surveillance/steps/riskfactor/en/ 10.2105/AJPH.2015.302962 PMC4695948 26696288

[21] World Health Organization WHO Study on global AGEing and adult health (SAGE). Available: https://www.who.int/healthinfo/sage/en/

[22] The World Bank World Bank country and lending groups (for fiscal year 2020), 2020 Available: https://datahelpdesk.worldbank.org/knowledgebase/articles/906519-world-bank-country-and-lending-groups

[23] United Nations Department of Economic and Social Affairs Population dynamics: world population prospects 2020, 2020 Available: https://population.un.org/wpp/

[24] Bull FC, Maslin TS, Armstrong T Global Physical Activity Questionnaire (GPAQ): nine country reliability and validity study. J Phys Act Health 2009;6:790–804. 10.1123/jpah.6.6.790 20101923

[25] Trinh OTH, Nguyen ND, van der Ploeg HP, et al Test-retest repeatability and relative validity of the Global Physical Activity Questionnaire in a developing country context. J Phys Act Health 2009;6 Suppl 1:S46–53. 10.1123/jpah.6.s1.s46 19998849

[26] Wanner M, Hartmann C, Pestoni G, et al Validation of the Global Physical Activity Questionnaire for self-administration in a European context. BMJ Open Sport Exerc Med 2017;3:e000206. 10.1136/bmjsem-2016-000206 28761703 PMC5530095

[27] Dugas LR, Bovet P, Forrester TE, et al Comparisons of intensity-duration patterns of physical activity in the US, Jamaica and 3 African countries. BMC Public Health 2014;14:882. 10.1186/1471-2458-14-882 25160601 PMC4168059

[28] Hoos T, Espinoza N, Marshall S, et al Validity of the Global Physical Activity Questionnaire (GPAQ) in adult Latinas. J Phys Act Health 2012;9:698–705. 10.1123/jpah.9.5.698 22733873 PMC3743722

[29] Hamilton NE, Ferry M ggtern: ternary diagrams using ggplot2. J Stat Softw 2018;87:1–17.

[30] Schöley J, Kashnitsky I tricolore: a flexible color scale for ternary compositions R package version 1.2.1, 2019 Available: https://CRAN.R-project.org/package=tricolore

[31] Guthold R, Louazani SA, Riley LM, et al Physical activity in 22 African countries: results from the World Health Organization STEPwise approach to chronic disease risk factor surveillance. Am J Prev Med 2011;41:52–60. 10.1016/j.amepre.2011.03.008 21665063

[32] Ng N, Hakimi M, Van Minh H, et al Prevalence of physical inactivity in nine rural indepth health and demographic surveillance systems in five Asian countries. Glob Health Action 2009;2. 10.3402/gha.v2i0.1985. [Epub ahead of print: 28 Sep 2009]. PMC2785136 20027261

[33] United Nations Sustainable Development Goals, 2020 Available: https://www.un.org/sustainabledevelopment/sustainable-development-goals/

[34] Bauman A, Allman-Farinelli M, Huxley R, et al Leisure-time physical activity alone may not be a sufficient public health approach to prevent obesity--a focus on China. Obes Rev 2008;9 Suppl 1:119–26. 10.1111/j.1467-789X.2007.00452.x 18307713

[35] Del Duca GF, Nahas MV, Garcia LMT, et al Active commuting reduces sociodemographic differences in adherence to recommendations derived from leisure-time physical activity among Brazilian adults. Public Health 2016;134:12–17. 10.1016/j.puhe.2016.01.016 26947312

[36] Coenen P, Huysmans MA, Holtermann A, et al Do highly physically active workers die early? A systematic review with meta-analysis of data from 193 696 participants. Br J Sports Med 2018;52:1320–6. 10.1136/bjsports-2017-098540 29760168

[37] Wijndaele K, DE Bourdeaudhuij I, Godino JG, et al Reliability and validity of a domain-specific last 7-d sedentary time questionnaire. Med Sci Sports Exerc 2014;46:1248–60. 10.1249/MSS.0000000000000214 24492633 PMC4047320

[38] Bui TV, Blizzard CL, Luong KN, et al Physical activity in Vietnam: estimates and measurement issues. PLoS One 2015;10:e0140941. 10.1371/journal.pone.0140941 26485044 PMC4618512

[39] Lindsay T, Westgate K, Wijndaele K, et al Descriptive epidemiology of physical activity energy expenditure in UK adults (the Fenland study). Int J Behav Nutr Phys Act 2019;16:126. 10.1186/s12966-019-0882-6 31818302 PMC6902569

[40] Barnett J, Nigg C, De Bourdeaudhuij I, et al Effect of item order on physical activity estimates using the IPAQ. Californian J Health Promot 2007;5:23–9. 10.32398/cjhp.v5i1.1799

[41] Roberts D, Townsend N, Foster C Use of new guidance to profile 'equivalent minutes' of aerobic physical activity for adults in England reveals gender, geographical, and socio-economic inequalities in meeting public health guidance: A cross-sectional study. Prev Med Rep 2016;4:50–60. 10.1016/j.pmedr.2016.05.009 27413661 PMC4929059

[42] Anjana RM, Pradeepa R, Das AK, et al Physical activity and inactivity patterns in India - results from the ICMR-INDIAB study (Phase-1) [ICMR-INDIAB-5]. Int J Behav Nutr Phys Act 2014;11:26. 10.1186/1479-5868-11-26 24571915 PMC3974063

[43] World Health Assembly WHA71.6 WHO global action plan on physical activity 2018–2030, 2018.

