# Supplementary figures and images for: Levels of domain-specific physical activity at work, in the household, for travel and for leisure among 327 789 adults from 104 countries
Source: Br J Sports Med. 2020 Nov 23;54(24):1488–97. doi: 10.1136/bjsports-2020-102601 (PMC7719912; doi:10.1136/bjsports-2020-102601)

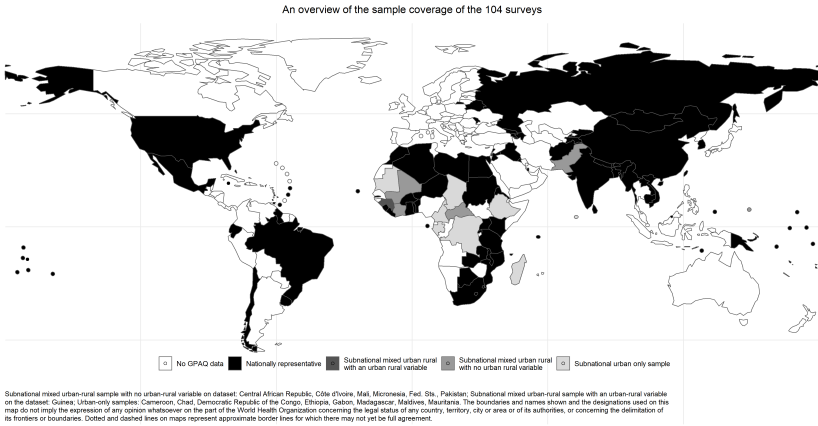

Supplement: Supplementary data [file bjsports-2020-102601supp002.pdf]

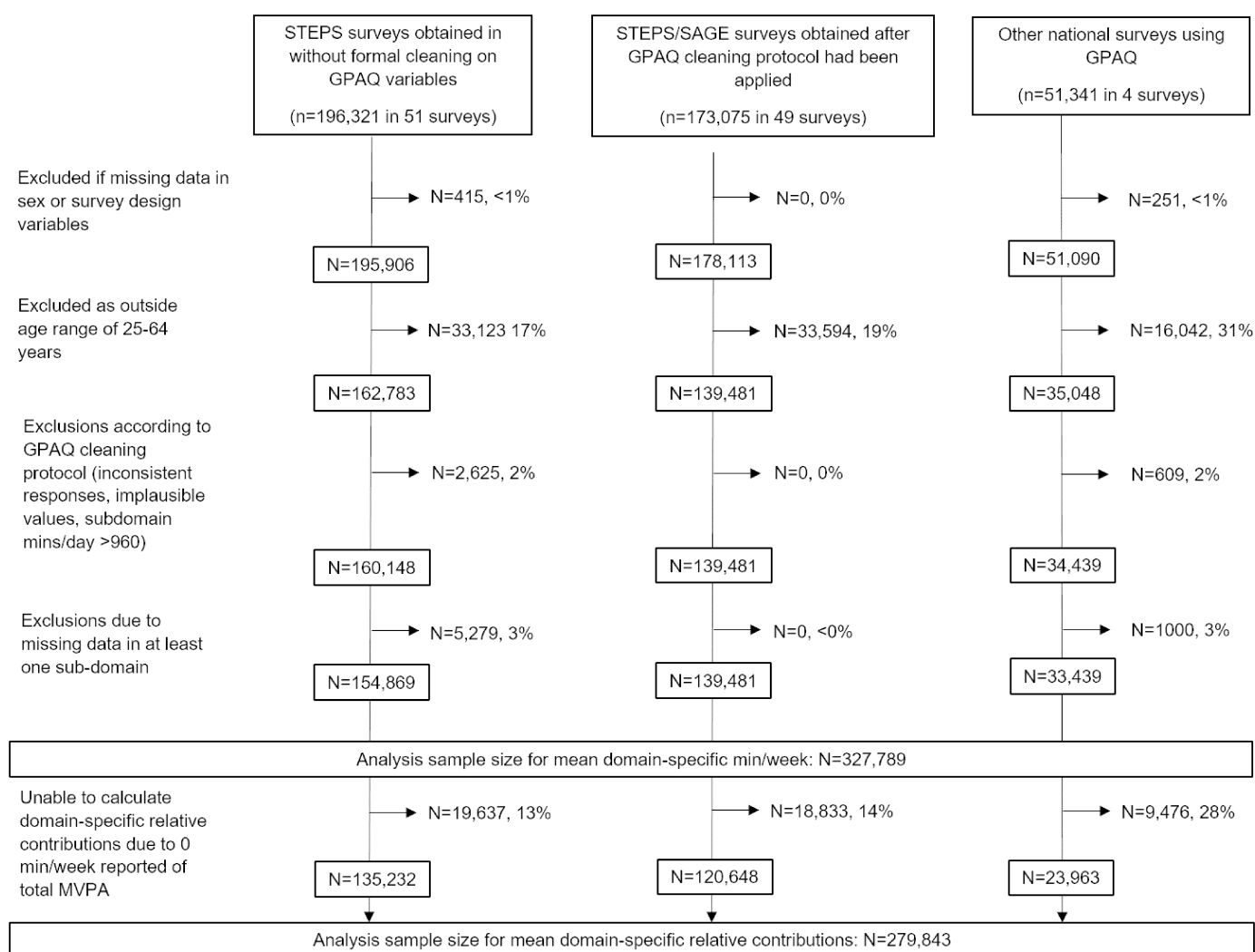

Supplement: Supplementary data [file bjsports-2020-102601supp005.pdf]

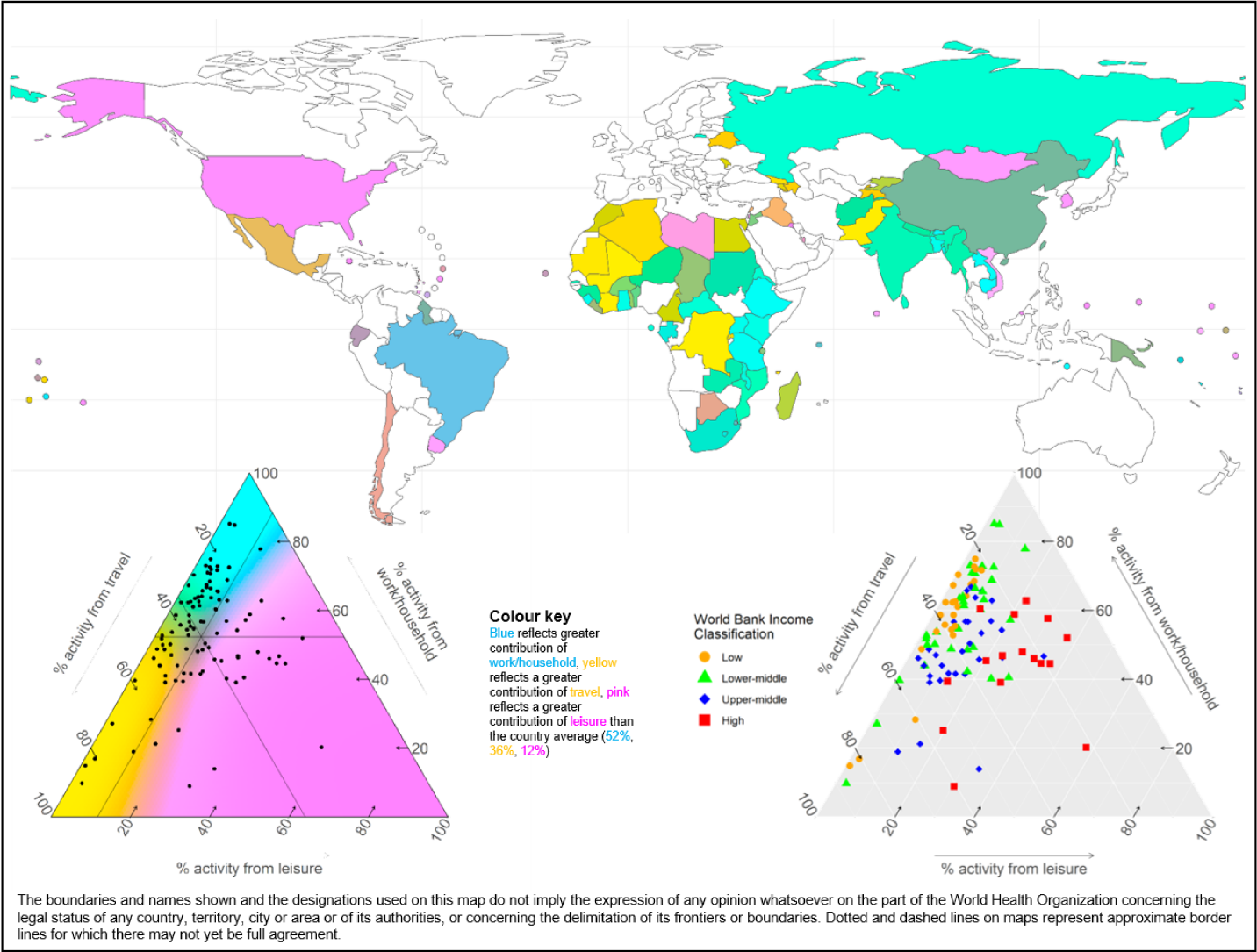

Supplement: Supplementary data [file bjsports-2020-102601supp007.pdf]
