# Supplementary material for: Levels of domain-specific physical activity at work, in the household, for travel and for leisure among 327 789 adults from 104 countries
Source: Br J Sports Med. 2020 Nov 23;54(24):1488–97. doi: 10.1136/bjsports-2020-102601 (PMC7719912; doi:10.1136/bjsports-2020-102601)
Supplement: Supplementary data [file bjsports-2020-102601supp003.pdf]

Supplementary File 2. Adjusting for urban-rural coverage in subnational samples

Overview of national and subnational survey samples

Of the 104 surveys, 14 were subnational. Of the subnational surveys, 8 were urban only, 6 were mixed urban-rural. Of the mixed urban-rural samples, 1 had an indicator variable to identify respondents as living in urban or rural area. The sample coverage and the presence of an indicator variable determined how the surveys were analysed as indicated in the flowchart below.

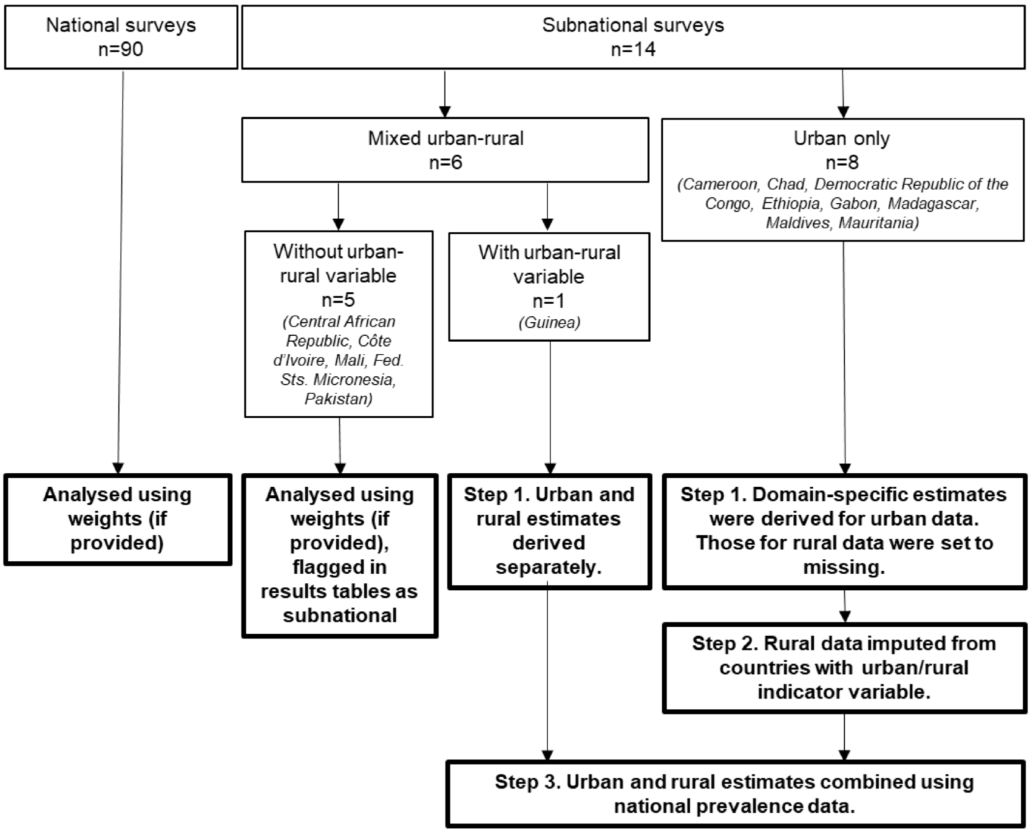

Overview of method to adjust subnational samples

The flowchart above shows that for the majority (9 of 14) of subnational surveys, measures were taken to adjust for urban-rural coverage. The 5 surveys with mixed urban-rural samples with no indicator variable were analysed with no adjustment.

The process for adjustment involved three steps: (1) deriving summary estimates of domain-specific MVPA separately for urban and rural individuals, (2) imputing rural values for urban only samples, (3) combining urban and rural estimates according to national urban-rural prevalence data.

The flowchart below provides more details on the data used in this process.

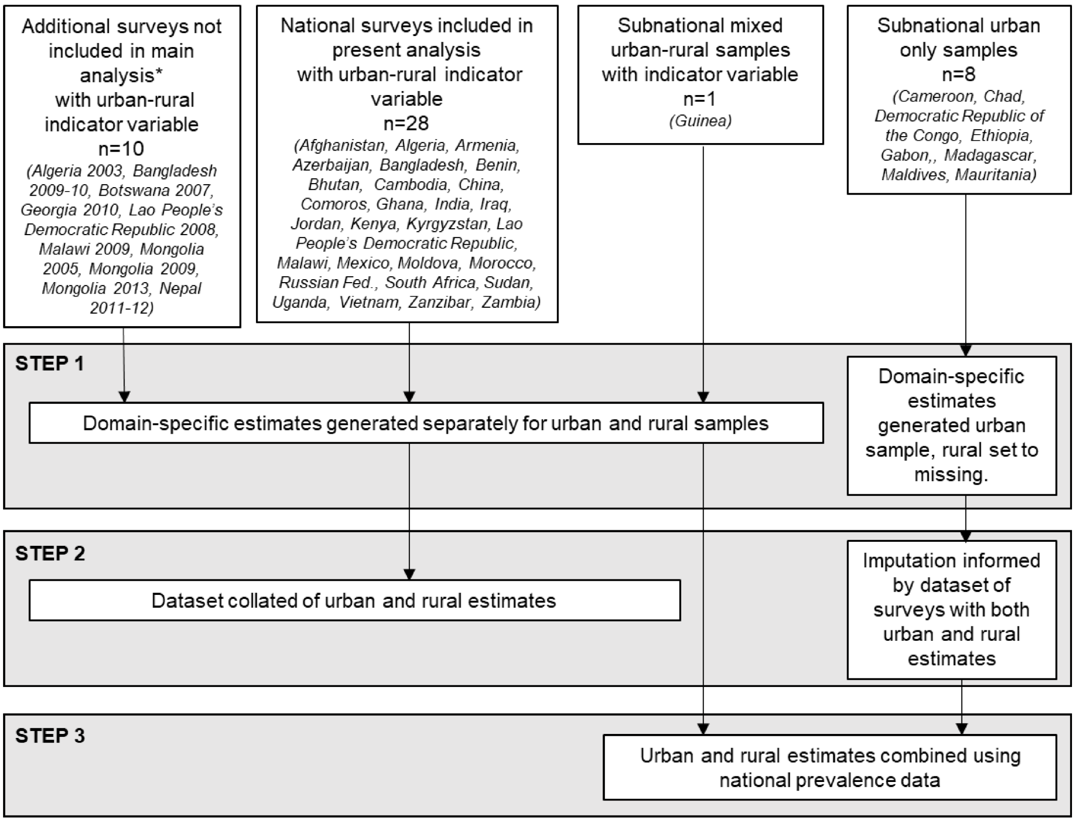

**Additional surveys include STEPS surveys (i.e. same methods) carried out in countries at an earlier time-point to those included in main analysis. We included these to provide more data to inform the imputations. Descriptive details of these surveys are included at the end of this Supplementary Methods section.*

The end goal of the 3-step process was to be able to populate the table below for each subnational survey, having taken into account the urban-rural distribution of the country.

**Demographic group**	**Mean minutes/week <u>including</u> those reporting 0 of total MVPA**			**Mean minutes/week <u>excluding</u> those reporting 0 of total MVPA**		
	**Work/household**	**Travel**	**Leisure**	**Work/household**	**Travel**	**Leisure**
Overall						
Male						
Female						
25-44 years						
45-64 years						

Step 1. Deriving domain-specific estimates for urban and rural samples separately

In Step 1, domain-specific estimates of MVPA were calculated for each sex, age group and urban-rural strata for all of the surveys detailed in the flowchart above. The table below illustrates the information derived for each survey. For the 8 urban-only samples, the rural categories were set to missing.

Sex	Age group	Urban/Rural	Mean minutes/week **including** those reporting 0 of total MVPA			Mean minutes/week **excluding** those reporting 0 of total MVPA		
			Work/ household	Travel	Leisure	Work/ household	Travel	Leisure
Male	25-44 years	Urban						
Male	25-44 years	Rural						
Male	45-64 years	Urban						
Male	45-64 years	Rural						
Female	25-44 years	Urban						
Female	25-44 years	Rural						
Female	45-64 years	Urban						
Female	45-64 years	Rural						

Step 2.

For each domain, the mean ratio of rural:urban was calculated amongst the 38 surveys with complete data. This was multiplied with the urban values in the 8 urban-only surveys. This process was done separately for each of the sex*age group strata and for each domain-specific estimate of MVPA.

We considered using ratios specific for countries of the same income strata to try to maximise the similarity between the data used for imputation and those that were being imputed. However, this would mean less data informing each imputation. As all countries involved in the imputation were from a range of low, lower-middle, and upper-middle income settings, we felt it was better to opt in favour of using the totality of the data.

Multiple imputation using chained equations was considered but with the high ratio of missing data to complete cases, and the clustered nature of the data, the model was very unstable. This method was preferable as it placed greater weight on the collected urban data.

Step 3.

Only the 9 subnational surveys were taken forward to step 3. Population data on urban-rural prevalence specific to the survey year were obtained from https://population.un.org/wup/DataQuery/ (see Table at end of Supplementary Methods 1 for data). Weighting the estimates according to these data, the urban and rural estimates were combined so the dataset contained information able to populate the following table:

Sex	Age group	Mean minutes/week **including** those reporting 0 of total MVPA			Mean minutes/week **excluding** those reporting 0 of total MVPA		
		Work/	Travel	Leisure	Work/	Travel	Leisure

		**household**			**household**		
Male	25-44 years						
Male	45-64 years						
Female	25-44 years						
Female	45-64 years						

Next, population data were obtained for the sex and age group breakdowns (https://population.un.org/wpp/Download/Standard/Population/; see Table at end of Supplementary Methods 1). Using these proportions, the four sex*age group estimates were combined to produce an overall country-level estimate, and sex and age stratified estimates.

The mean minutes/week of MVPA including those reporting 0 of total MVPA were used in Figure 1. The mean minutes/week of MVPA excluding those reporting 0 of total MVPA were used to generate relative proportions that were included in Figures 2 and 3, Table 2 and Supplementary Files 8 and 9.

Details of the 10 additional STEPS surveys used to inform the imputation process

Country	Date of fieldwork	Months of fieldwork	Season of fieldwork	World Health Organization region	World Bank Income Classification	Sample
Algeria	2003	Sep to Oct	Autumn, dry	Africa	UM	Sub-national including the wilayas of Sérif and Mostaganem
Bangladesh	2009-10	Nov to Apr	Dry winter and spring	South-East Asia	LM	Nationally representative
Botswana	2007	Mar to May	Autumn	Africa	UM	Nationally representative
Georgia	2010	Aug to Dec	Autumn, winter	Europe	UM	Nationally representative
Lao PDR	2008	Jan to Apr	Dry	Western Pacific	LM	Sub-national including Vientiane (capital)
Malawi	2009	Jun to Sep	Dry	Africa	L	Nationally representative
Mongolia	2005	May to Oct	Summer, autumn	Western Pacific	LM	Nationally representative
Mongolia	2009	Oct to Dec	Autumn, winter	Western Pacific	LM	Nationally representative
Mongolia	2013	May to Jun	Spring	Western Pacific	LM	Nationally representative
Nepal	2012-13	Jul to Jun	All year	South-East Asia	L	Nationally representative

UM=upper-middle, LM=lower-middle, L=low.

The urban and rural prevalence data according to the survey year for the 9 subnational countries for which adjustments were made

Country and year of survey	Urban population (thousands)	Rural population (thousands)	Urban %	Rural %
Cameroon 2003	7,850	8696	47	53
Chad 2008	2,438	8,695	22	78
Dem. Rep. Congo 2005	20,521	34,230	37	63
Ethiopia 2006	12,536	66,315	16	84
Gabon 2009	1,348	239	85	15
Guinea 2009	3,525	7,032	33	67
Madagascar 2005	5,284	13,053	29	71
Maldives 2011	138	237	37	63
Mauritania 2006	1,385	1,836	43	57

Age and sex specific population data used to combined the estimates for the 9 subnational surveys for which adjustments were made

	Males				Females			
	25-44		45-64		25-44		45-64	
	Number (1000s)	% of total	Number (1000s)	% of total	Number (1000s)	% of total	Number (1000s)	% of total
Cameroon 2003	1989.7	35.2	801.0	14.2	1998.7	35.4	862.1	15.3
Chad 2008	1226.8	35.4	486.0	14.0	1222.7	35.3	527.3	15.2
Dem. Rep. Congo 2005	6032.6	34.2	2643.4	15.0	6122.8	34.7	2850.3	16.1
Ethiopia 2006	8050.3	33.9	3579.3	15.1	8222.7	34.6	3899.0	16.4
Gabon 2009	235.9	37.8	87.5	14.0	212.5	34.0	88.7	14.2
Guinea 2009	921.4	30.0	395.3	12.9	1156.5	37.7	595.8	19.4
Madagascar 2005	2139.1	35.3	856.6	14.1	2158.9	35.6	912.4	15.0
Maldives 2011	66.9	40.5	24.1	14.6	53.0	32.0	21.4	12.9
Mauritania 2006	360.8	35.1	141.6	13.8	363.9	35.4	161.5	15.7
