# Supplementary material for: Levels of domain-specific physical activity at work, in the household, for travel and for leisure among 327 789 adults from 104 countries
Source: Br J Sports Med. 2020 Nov 23;54(24):1488–97. doi: 10.1136/bjsports-2020-102601 (PMC7719912; doi:10.1136/bjsports-2020-102601)
Supplement: Supplementary data [file bjsports-2020-102601supp006.pdf]

Scaled density distributions of the mean weekly minutes and relative contributions of domain-specific MVPA to total MVPA

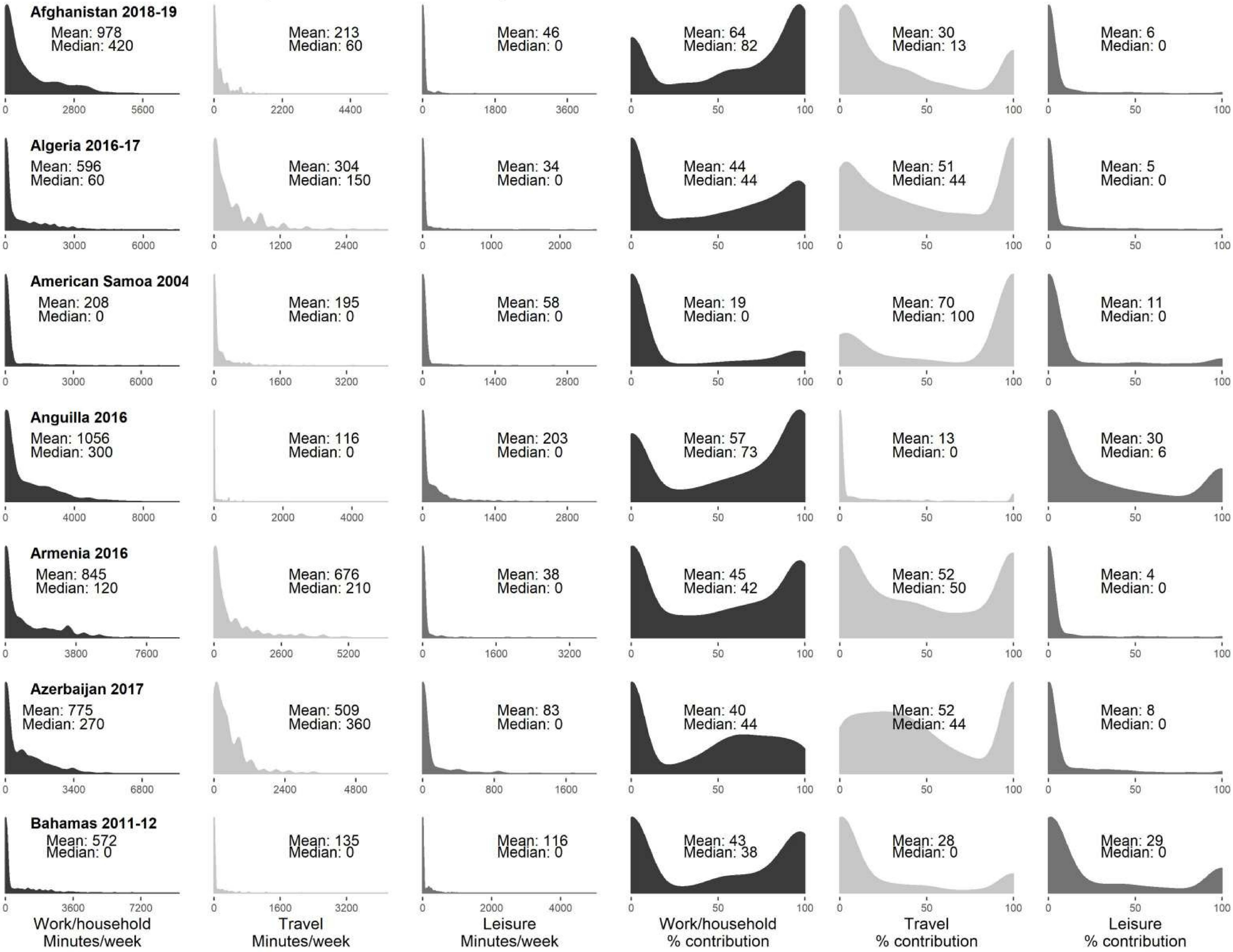

The first three panels display the scaled density distributions for the reported weekly minutes in the work/household, travel, and leisure domains respectively. The second three panels diplay the scaled density distributions for the relative contributions of the domains to total MVPA (requires at least 1 minute/week of MVPA across any domain to be reported to have non-zero denominator). Cameroon, Central African Republic, Chad, Côte d'Ivoire, Democratic Republic of the Congo, Ethiopia, Gabon, Guinea, Madagascar, Maldives, Mali, Mauritania, Micronesia Fed. Sts., and Pakistan are subnational surveys and no adjustment has been made to the data presented in this figure.

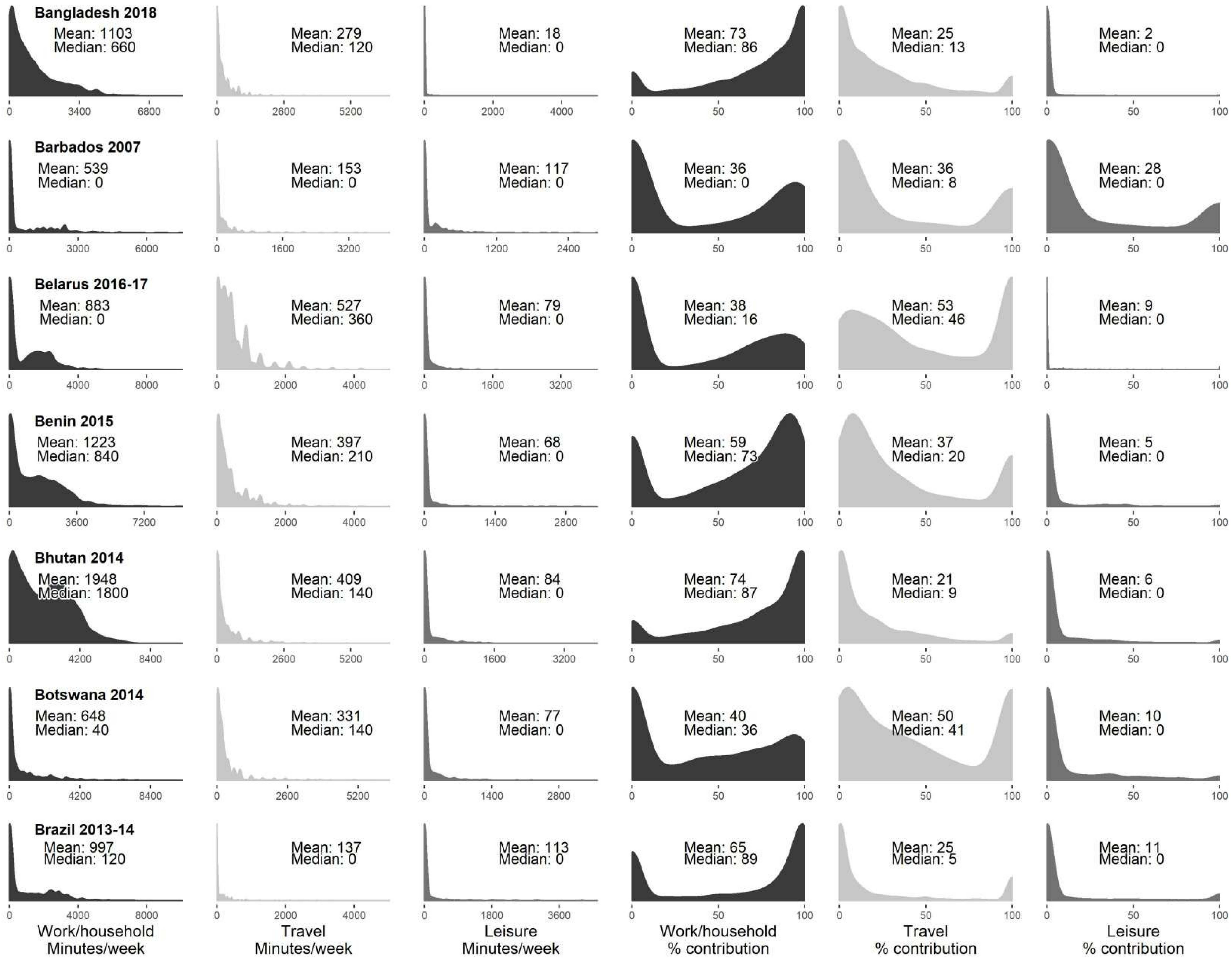
Bangladesh 2018
Mean: 1103
Median: 660
Mean: 279
Median: 120
Mean: 18
Median: 0
Mean: 73
Median: 86
Mean: 25
Median: 13
Mean: 2
Median: 0
Barbados 2007
Mean: 539
Median: 0
Mean: 153
Median: 0
Mean: 117
Median: 0
Mean: 36
Median: 0
Mean: 36
Median: 8
Mean: 28
Median: 0
Belarus 2016-17
Mean: 883
Median: 0
Mean: 527
Median: 360
Mean: 79
Median: 0
Mean: 38
Median: 16
Mean: 53
Median: 46
Mean: 9
Median: 0
Benin 2015
Mean: 1223
Median: 840
Mean: 397
Median: 210
Mean: 68
Median: 0
Mean: 59
Median: 73
Mean: 37
Median: 20
Mean: 5
Median: 0
Bhutan 2014
Mean: 1948
Median: 1800
Mean: 409
Median: 140
Mean: 84
Median: 0
Mean: 74
Median: 87
Mean: 21
Median: 9
Mean: 6
Median: 0
Botswana 2014
Mean: 648
Median: 40
Mean: 331
Median: 140
Mean: 77
Median: 0
Mean: 40
Median: 36
Mean: 50
Median: 41
Mean: 10
Median: 0
Brazil 2013-14
Mean: 997
Median: 120
Mean: 137
Median: 0
Mean: 113
Median: 0
Mean: 65
Median: 89
Mean: 25
Median: 5
Mean: 11
Median: 0
Work/household
Minutes/week
Travel
Minutes/week
Leisure
Minutes/week
Work/household
% contribution
Travel
% contribution
Leisure
% contribution

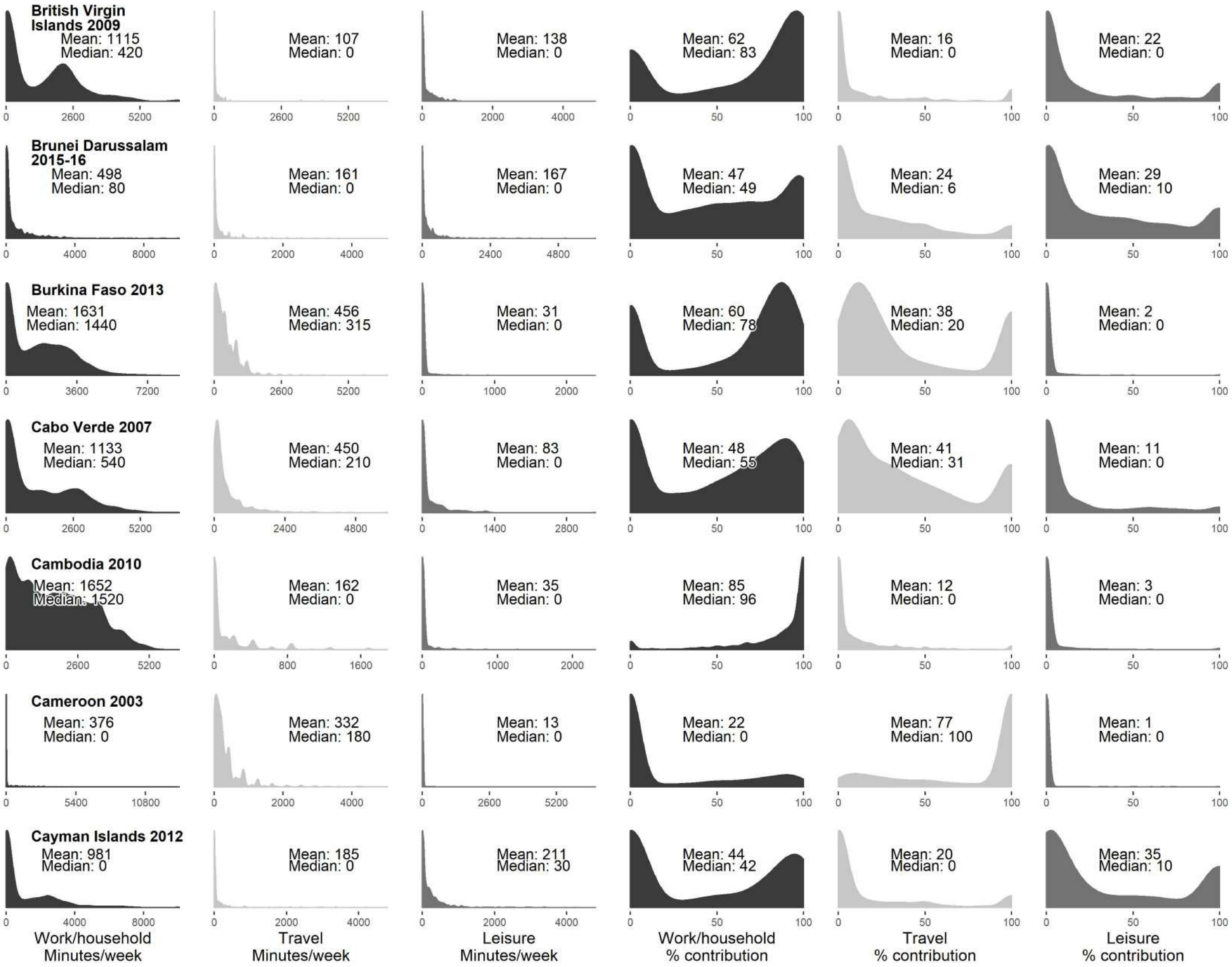
British Virgin Islands 2009
Mean: 1115
Median: 420
Mean: 107
Median: 0
Mean: 138
Median: 0
Mean: 62
Median: 83
Mean: 16
Median: 0
Mean: 22
Median: 0
Brunei Darussalam 2015-16
Mean: 498
Median: 80
Mean: 161
Median: 0
Mean: 167
Median: 0
Mean: 47
Median: 49
Mean: 24
Median: 6
Mean: 29
Median: 10
Burkina Faso 2013
Mean: 1631
Median: 1440
Mean: 456
Median: 315
Mean: 31
Median: 0
Mean: 60
Median: 78
Mean: 38
Median: 20
Mean: 2
Median: 0
Cabo Verde 2007
Mean: 1133
Median: 540
Mean: 450
Median: 210
Mean: 83
Median: 0
Mean: 48
Median: 55
Mean: 41
Median: 31
Mean: 11
Median: 0
Cambodia 2010
Mean: 1652
Median: 1520
Mean: 162
Median: 0
Mean: 35
Median: 0
Mean: 85
Median: 96
Mean: 12
Median: 0
Mean: 3
Median: 0
Cameroon 2003
Mean: 376
Median: 0
Mean: 332
Median: 180
Mean: 13
Median: 0
Mean: 22
Median: 0
Mean: 77
Median: 100
Mean: 1
Median: 0
Cayman Islands 2012
Mean: 981
Median: 0
Mean: 185
Median: 0
Mean: 211
Median: 30
Mean: 44
Median: 42
Mean: 20
Median: 0
Mean: 35
Median: 10
Work/household Minutes/week
Travel Minutes/week
Leisure Minutes/week
Work/household % contribution
Travel % contribution
Leisure % contribution

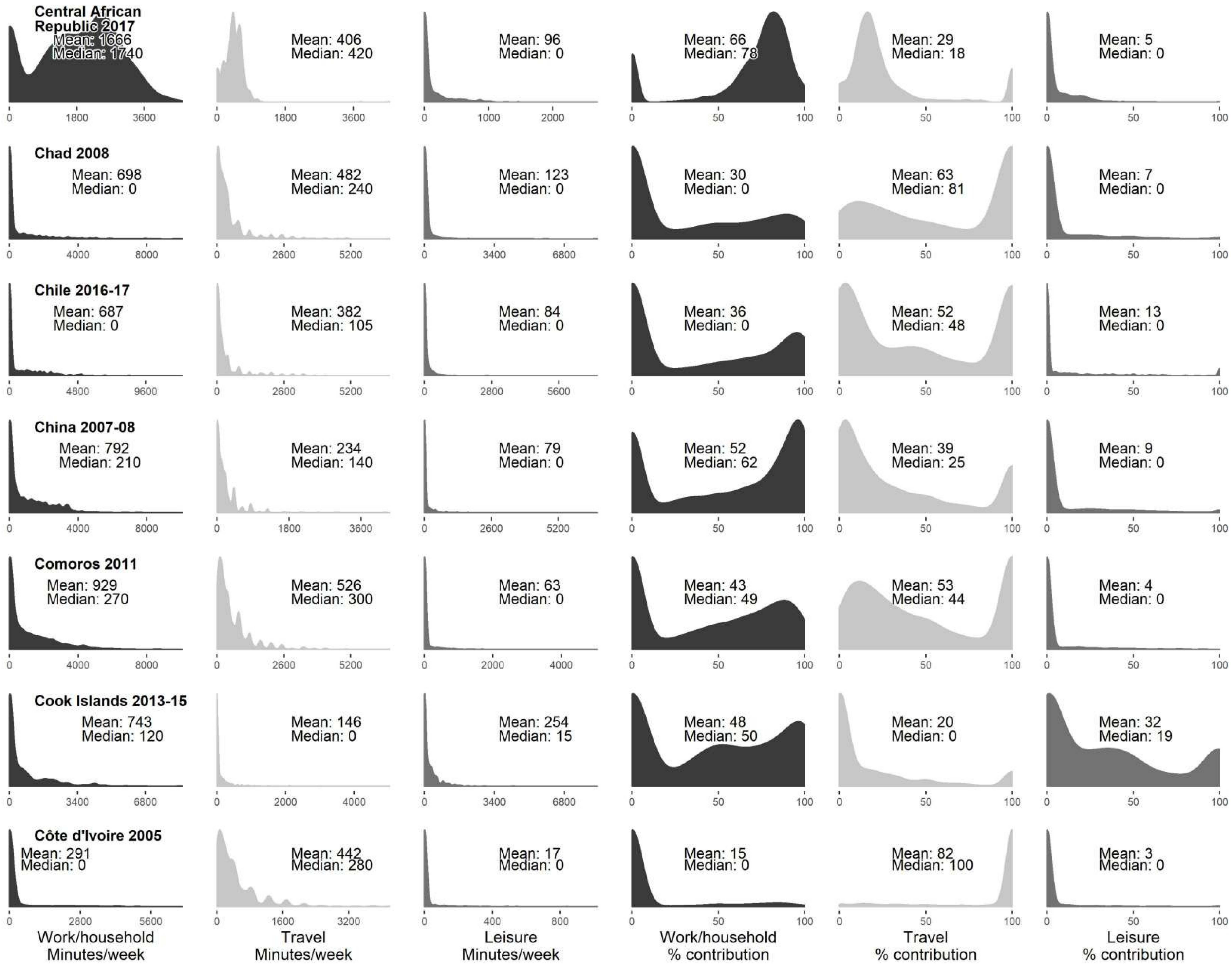
Central African Republic 2017
Mean: 1666
Median: 1740
Mean: 406
Median: 420
Mean: 96
Median: 0
Mean: 66
Median: 78
Mean: 29
Median: 18
Mean: 5
Median: 0
Chad 2008
Mean: 698
Median: 0
Mean: 482
Median: 240
Mean: 123
Median: 0
Mean: 30
Median: 0
Mean: 63
Median: 81
Mean: 7
Median: 0
Chile 2016-17
Mean: 687
Median: 0
Mean: 382
Median: 105
Mean: 84
Median: 0
Mean: 36
Median: 0
Mean: 52
Median: 48
Mean: 13
Median: 0
China 2007-08
Mean: 792
Median: 210
Mean: 234
Median: 140
Mean: 79
Median: 0
Mean: 52
Median: 62
Mean: 39
Median: 25
Mean: 9
Median: 0
Comoros 2011
Mean: 929
Median: 270
Mean: 526
Median: 300
Mean: 63
Median: 0
Mean: 43
Median: 49
Mean: 53
Median: 44
Mean: 4
Median: 0
Cook Islands 2013-15
Mean: 743
Median: 120
Mean: 146
Median: 0
Mean: 254
Median: 15
Mean: 48
Median: 50
Mean: 20
Median: 0
Mean: 32
Median: 19
Côte d'Ivoire 2005
Mean: 291
Median: 0
Mean: 442
Median: 280
Mean: 17
Median: 0
Mean: 15
Median: 0
Mean: 82
Median: 100
Mean: 3
Median: 0
Work/household Minutes/week
Travel Minutes/week
Leisure Minutes/week
Work/household % contribution
Travel % contribution
Leisure % contribution

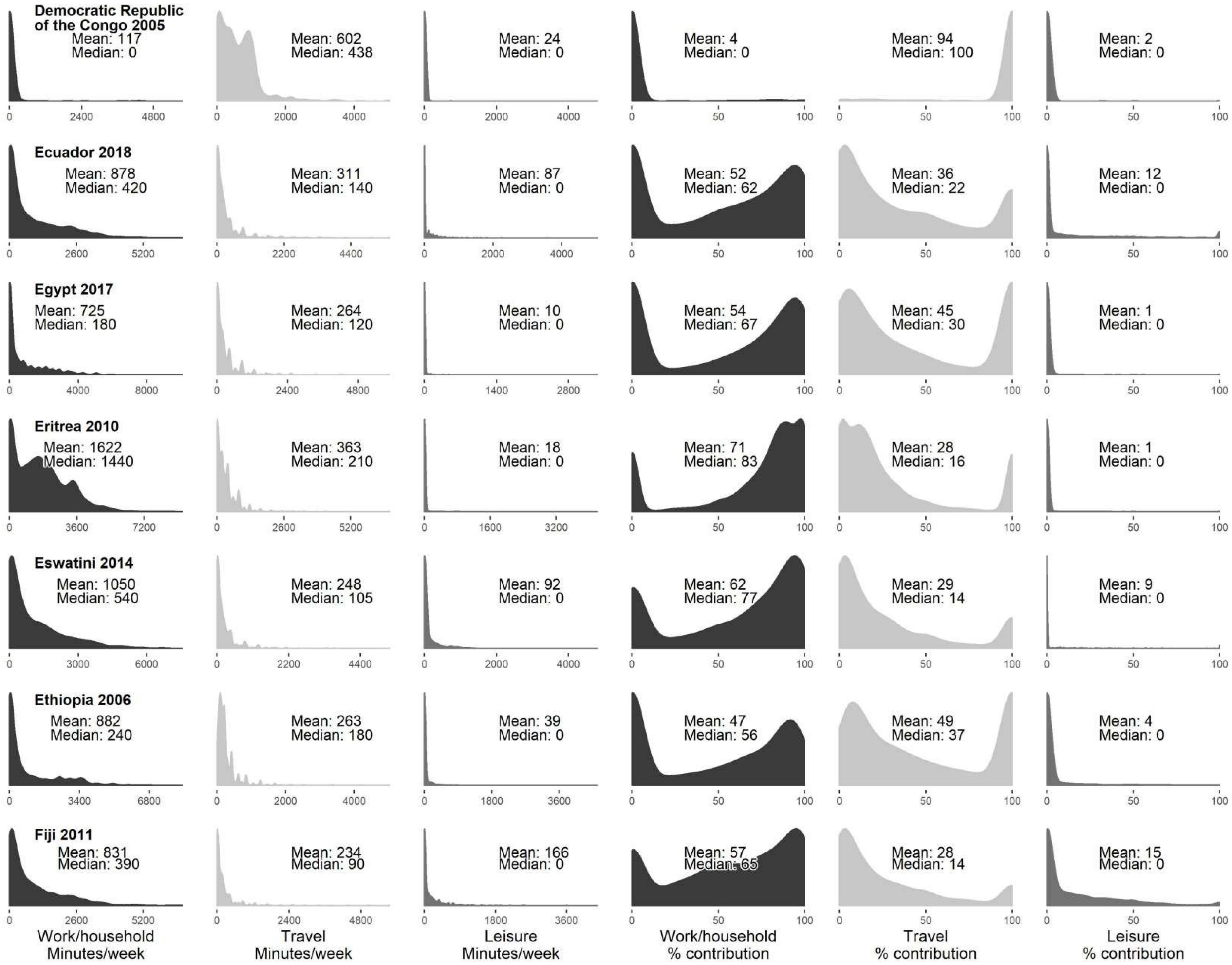
Democratic Republic of the Congo 2005
Mean: 117
Median: 0
Mean: 602
Median: 438
Mean: 24
Median: 0
Mean: 4
Median: 0
Mean: 94
Median: 100
Mean: 2
Median: 0
Ecuador 2018
Mean: 878
Median: 420
Mean: 311
Median: 140
Mean: 87
Median: 0
Mean: 52
Median: 62
Mean: 36
Median: 22
Mean: 12
Median: 0
Egypt 2017
Mean: 725
Median: 180
Mean: 264
Median: 120
Mean: 10
Median: 0
Mean: 54
Median: 67
Mean: 45
Median: 30
Mean: 1
Median: 0
Eritrea 2010
Mean: 1622
Median: 1440
Mean: 363
Median: 210
Mean: 18
Median: 0
Mean: 71
Median: 83
Mean: 28
Median: 16
Mean: 1
Median: 0
Eswatini 2014
Mean: 1050
Median: 540
Mean: 248
Median: 105
Mean: 92
Median: 0
Mean: 62
Median: 77
Mean: 29
Median: 14
Mean: 9
Median: 0
Ethiopia 2006
Mean: 882
Median: 240
Mean: 263
Median: 180
Mean: 39
Median: 0
Mean: 47
Median: 56
Mean: 49
Median: 37
Mean: 4
Median: 0
Fiji 2011
Mean: 831
Median: 390
Mean: 234
Median: 90
Mean: 166
Median: 0
Mean: 57
Median: 65
Mean: 28
Median: 14
Mean: 15
Median: 0
Work/household Minutes/week
Travel Minutes/week
Leisure Minutes/week
Work/household % contribution
Travel % contribution
Leisure % contribution

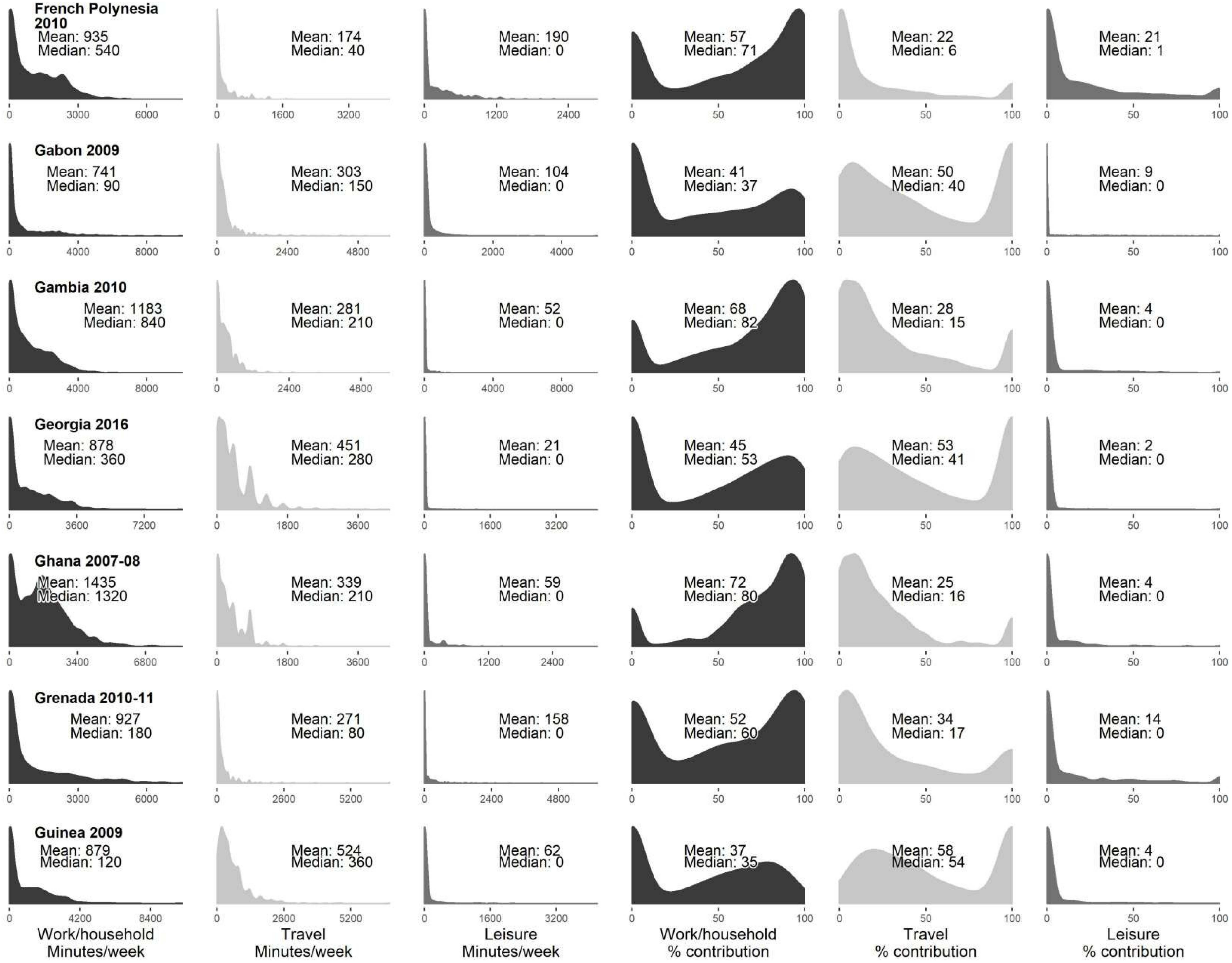
French Polynesia 2010
Mean: 935
Median: 540
Mean: 174
Median: 40
Mean: 190
Median: 0
Mean: 57
Median: 71
Mean: 22
Median: 6
Mean: 21
Median: 1
Gabon 2009
Mean: 741
Median: 90
Mean: 303
Median: 150
Mean: 104
Median: 0
Mean: 41
Median: 37
Mean: 50
Median: 40
Mean: 9
Median: 0
Gambia 2010
Mean: 1183
Median: 840
Mean: 281
Median: 210
Mean: 52
Median: 0
Mean: 68
Median: 82
Mean: 28
Median: 15
Mean: 4
Median: 0
Georgia 2016
Mean: 878
Median: 360
Mean: 451
Median: 280
Mean: 21
Median: 0
Mean: 45
Median: 53
Mean: 53
Median: 41
Mean: 2
Median: 0
Ghana 2007-08
Mean: 1435
Median: 1320
Mean: 339
Median: 210
Mean: 59
Median: 0
Mean: 72
Median: 80
Mean: 25
Median: 16
Mean: 4
Median: 0
Grenada 2010-11
Mean: 927
Median: 180
Mean: 271
Median: 80
Mean: 158
Median: 0
Mean: 52
Median: 60
Mean: 34
Median: 17
Mean: 14
Median: 0
Guinea 2009
Mean: 879
Median: 120
Mean: 524
Median: 360
Mean: 62
Median: 0
Mean: 37
Median: 35
Mean: 58
Median: 54
Mean: 4
Median: 0
Work/household Minutes/week
Travel Minutes/week
Leisure Minutes/week
Work/household % contribution
Travel % contribution
Leisure % contribution

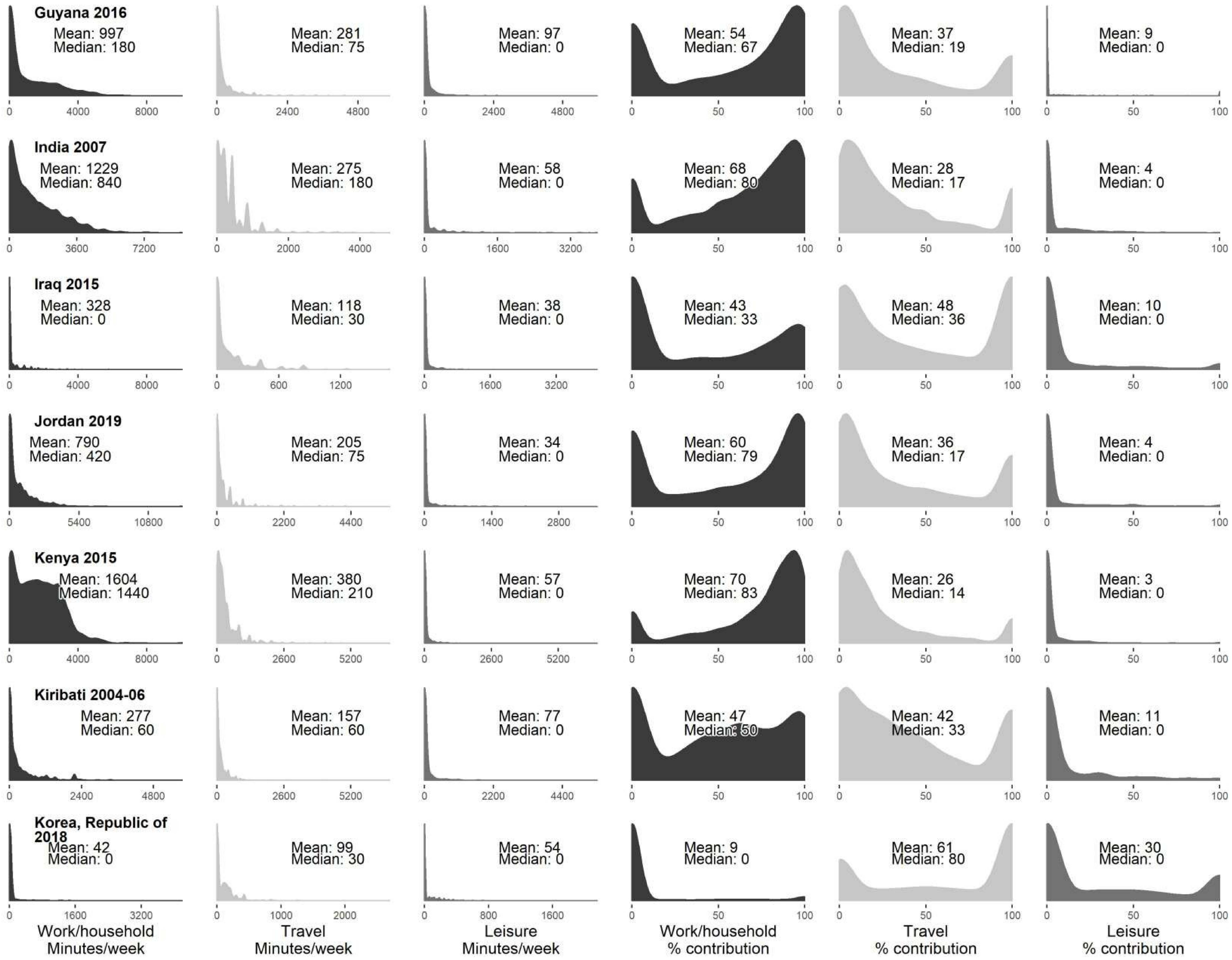
Guyana 2016
Mean: 997
Median: 180
Mean: 281
Median: 75
Mean: 97
Median: 0
Mean: 54
Median: 67
Mean: 37
Median: 19
Mean: 9
Median: 0
India 2007
Mean: 1229
Median: 840
Mean: 275
Median: 180
Mean: 58
Median: 0
Mean: 68
Median: 80
Mean: 28
Median: 17
Mean: 4
Median: 0
Iraq 2015
Mean: 328
Median: 0
Mean: 118
Median: 30
Mean: 38
Median: 0
Mean: 43
Median: 33
Mean: 48
Median: 36
Mean: 10
Median: 0
Jordan 2019
Mean: 790
Median: 420
Mean: 205
Median: 75
Mean: 34
Median: 0
Mean: 60
Median: 79
Mean: 36
Median: 17
Mean: 4
Median: 0
Kenya 2015
Mean: 1604
Median: 1440
Mean: 380
Median: 210
Mean: 57
Median: 0
Mean: 70
Median: 83
Mean: 26
Median: 14
Mean: 3
Median: 0
Kiribati 2004-06
Mean: 277
Median: 60
Mean: 157
Median: 60
Mean: 77
Median: 0
Mean: 47
Median: 50
Mean: 42
Median: 33
Mean: 11
Median: 0
Korea, Republic of 2018
Mean: 42
Median: 0
Mean: 99
Median: 30
Mean: 54
Median: 0
Mean: 9
Median: 0
Mean: 61
Median: 80
Mean: 30
Median: 0
Work/household Minutes/week
Travel Minutes/week
Leisure Minutes/week
Work/household % contribution
Travel % contribution
Leisure % contribution

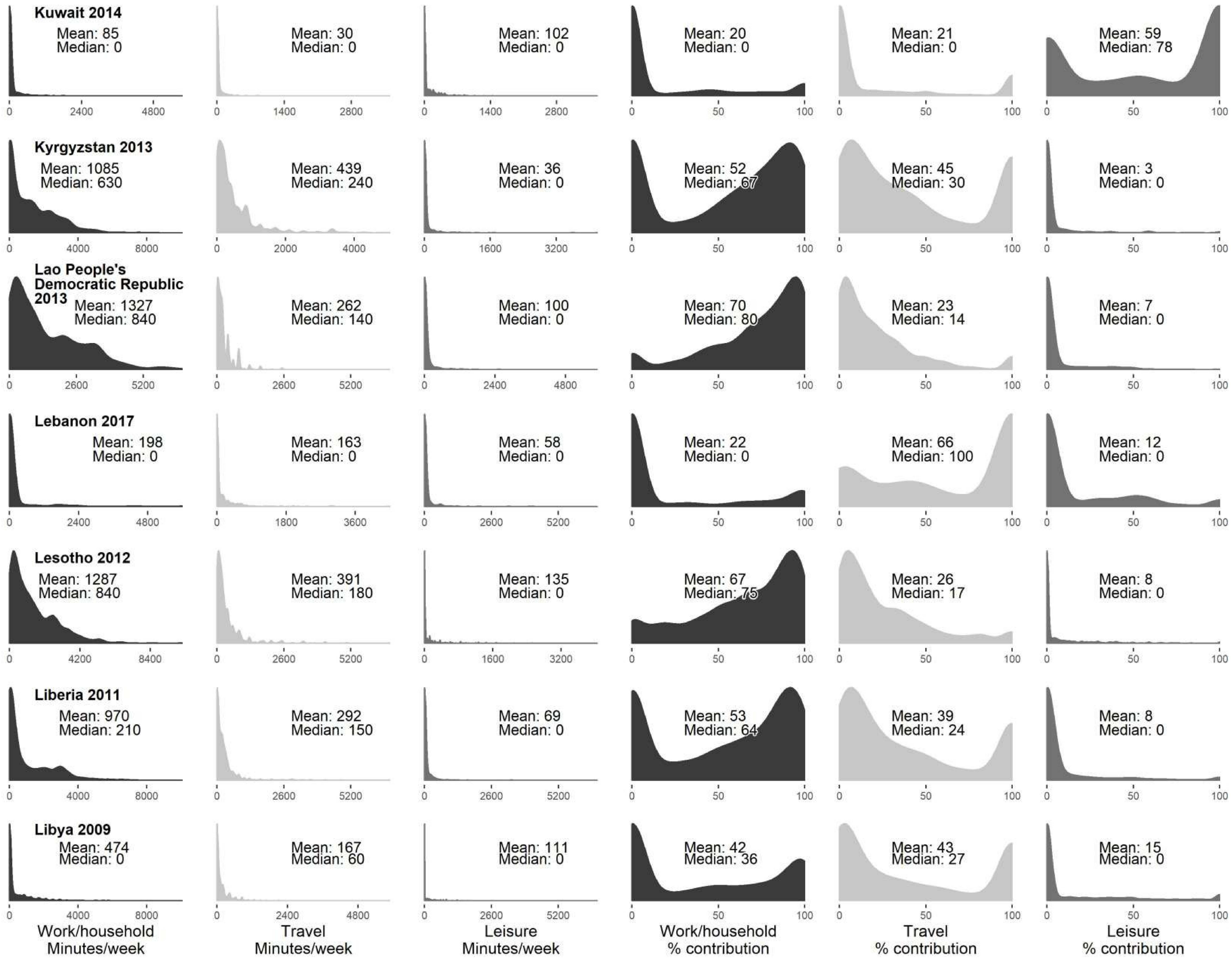
Kuwait 2014
Mean: 85
Median: 0
Mean: 30
Median: 0
Mean: 102
Median: 0
Mean: 20
Median: 0
Mean: 21
Median: 0
Mean: 59
Median: 78
Kyrgyzstan 2013
Mean: 1085
Median: 630
Mean: 439
Median: 240
Mean: 36
Median: 0
Mean: 52
Median: 67
Mean: 45
Median: 30
Mean: 3
Median: 0
Lao People's Democratic Republic 2013
Mean: 1327
Median: 840
Mean: 262
Median: 140
Mean: 100
Median: 0
Mean: 70
Median: 80
Mean: 23
Median: 14
Mean: 7
Median: 0
Lebanon 2017
Mean: 198
Median: 0
Mean: 163
Median: 0
Mean: 58
Median: 0
Mean: 22
Median: 0
Mean: 66
Median: 100
Mean: 12
Median: 0
Lesotho 2012
Mean: 1287
Median: 840
Mean: 391
Median: 180
Mean: 135
Median: 0
Mean: 67
Median: 75
Mean: 26
Median: 17
Mean: 8
Median: 0
Liberia 2011
Mean: 970
Median: 210
Mean: 292
Median: 150
Mean: 69
Median: 0
Mean: 53
Median: 64
Mean: 39
Median: 24
Mean: 8
Median: 0
Libya 2009
Mean: 474
Median: 0
Mean: 167
Median: 60
Mean: 111
Median: 0
Mean: 42
Median: 36
Mean: 43
Median: 27
Mean: 15
Median: 0
Work/household Minutes/week
Travel Minutes/week
Leisure Minutes/week
Work/household % contribution
Travel % contribution
Leisure % contribution

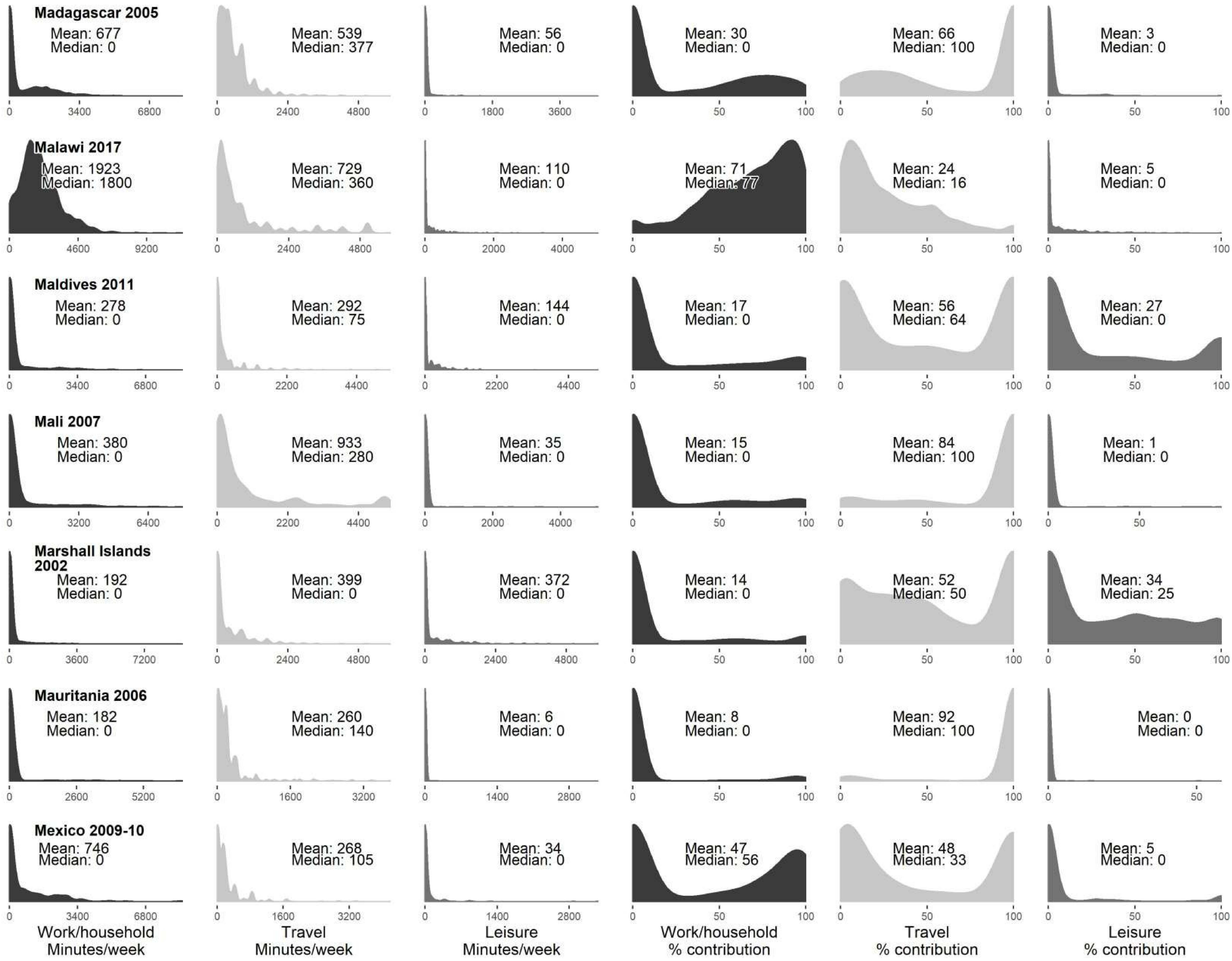
Madagascar 2005
Mean: 677 Median: 0
Mean: 539 Median: 377
Mean: 56 Median: 0
Mean: 30 Median: 0
Mean: 66 Median: 100
Mean: 3 Median: 0
Malawi 2017
Mean: 1923 Median: 1800
Mean: 729 Median: 360
Mean: 110 Median: 0
Mean: 71 Median: 77
Mean: 24 Median: 16
Mean: 5 Median: 0
Maldives 2011
Mean: 278 Median: 0
Mean: 292 Median: 75
Mean: 144 Median: 0
Mean: 17 Median: 0
Mean: 56 Median: 64
Mean: 27 Median: 0
Mali 2007
Mean: 380 Median: 0
Mean: 933 Median: 280
Mean: 35 Median: 0
Mean: 15 Median: 0
Mean: 84 Median: 100
Mean: 1 Median: 0
Marshall Islands 2002
Mean: 192 Median: 0
Mean: 399 Median: 0
Mean: 372 Median: 0
Mean: 14 Median: 0
Mean: 52 Median: 50
Mean: 34 Median: 25
Mauritania 2006
Mean: 182 Median: 0
Mean: 260 Median: 140
Mean: 6 Median: 0
Mean: 8 Median: 0
Mean: 92 Median: 100
Mean: 0 Median: 0
Mexico 2009-10
Mean: 746 Median: 0
Mean: 268 Median: 105
Mean: 34 Median: 0
Mean: 47 Median: 56
Mean: 48 Median: 33
Mean: 5 Median: 0
Work/household Minutes/week
Travel Minutes/week
Leisure Minutes/week
Work/household % contribution
Travel % contribution
Leisure % contribution

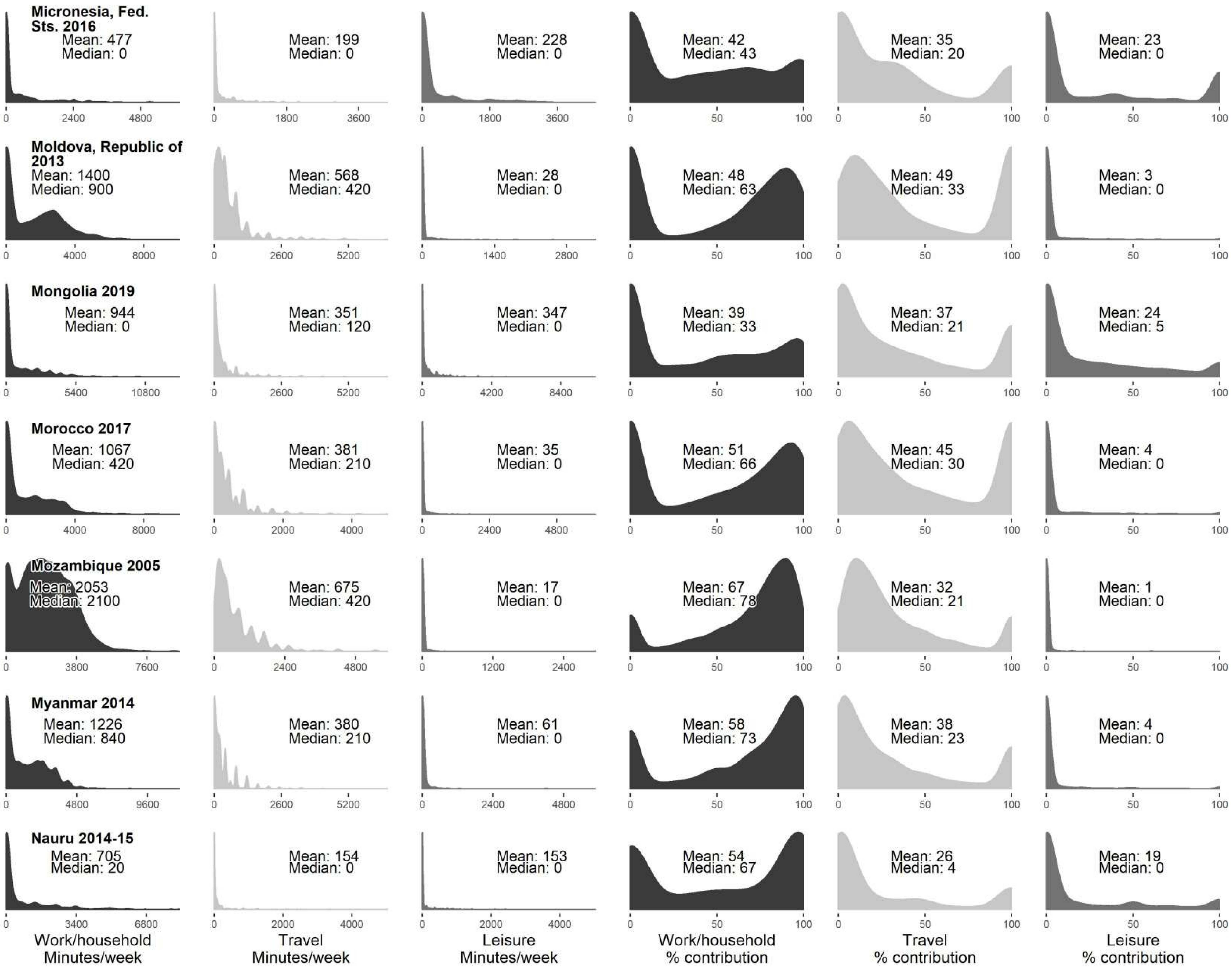
Micronesia, Fed. Sts. 2016
Mean: 477
Median: 0
Mean: 199
Median: 0
Mean: 228
Median: 0
Mean: 42
Median: 43
Mean: 35
Median: 20
Mean: 23
Median: 0
Moldova, Republic of 2013
Mean: 1400
Median: 900
Mean: 568
Median: 420
Mean: 28
Median: 0
Mean: 48
Median: 63
Mean: 49
Median: 33
Mean: 3
Median: 0
Mongolia 2019
Mean: 944
Median: 0
Mean: 351
Median: 120
Mean: 347
Median: 0
Mean: 39
Median: 33
Mean: 37
Median: 21
Mean: 24
Median: 5
Morocco 2017
Mean: 1067
Median: 420
Mean: 381
Median: 210
Mean: 35
Median: 0
Mean: 51
Median: 66
Mean: 45
Median: 30
Mean: 4
Median: 0
Mozambique 2005
Mean: 2053
Median: 2100
Mean: 675
Median: 420
Mean: 17
Median: 0
Mean: 67
Median: 78
Mean: 32
Median: 21
Mean: 1
Median: 0
Myanmar 2014
Mean: 1226
Median: 840
Mean: 380
Median: 210
Mean: 61
Median: 0
Mean: 58
Median: 73
Mean: 38
Median: 23
Mean: 4
Median: 0
Nauru 2014-15
Mean: 705
Median: 20
Mean: 154
Median: 0
Mean: 153
Median: 0
Mean: 54
Median: 67
Mean: 26
Median: 4
Mean: 19
Median: 0
Work/household Minutes/week
Travel Minutes/week
Leisure Minutes/week
Work/household % contribution
Travel % contribution
Leisure % contribution

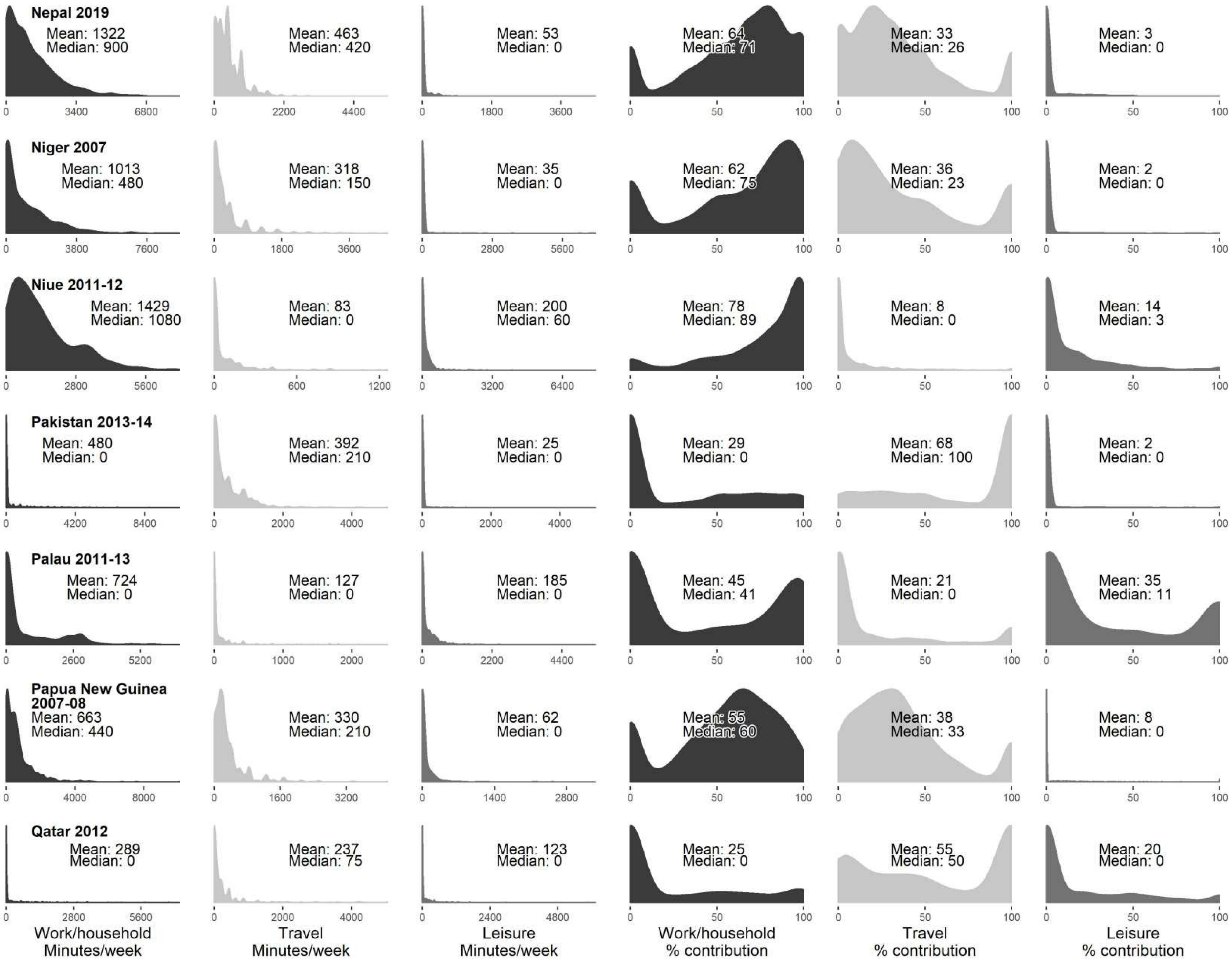
Nepal 2019
Mean: 1322
Median: 900
Mean: 463
Median: 420
Mean: 53
Median: 0
Mean: 64
Median: 71
Mean: 33
Median: 26
Mean: 3
Median: 0
Niger 2007
Mean: 1013
Median: 480
Mean: 318
Median: 150
Mean: 35
Median: 0
Mean: 62
Median: 75
Mean: 36
Median: 23
Mean: 2
Median: 0
Niue 2011-12
Mean: 1429
Median: 1080
Mean: 83
Median: 0
Mean: 200
Median: 60
Mean: 78
Median: 89
Mean: 8
Median: 0
Mean: 14
Median: 3
Pakistan 2013-14
Mean: 480
Median: 0
Mean: 392
Median: 210
Mean: 25
Median: 0
Mean: 29
Median: 0
Mean: 68
Median: 100
Mean: 2
Median: 0
Palau 2011-13
Mean: 724
Median: 0
Mean: 127
Median: 0
Mean: 185
Median: 0
Mean: 45
Median: 41
Mean: 21
Median: 0
Mean: 35
Median: 11
Papua New Guinea 2007-08
Mean: 663
Median: 440
Mean: 330
Median: 210
Mean: 62
Median: 0
Mean: 55
Median: 60
Mean: 38
Median: 33
Mean: 8
Median: 0
Qatar 2012
Mean: 289
Median: 0
Mean: 237
Median: 75
Mean: 123
Median: 0
Mean: 25
Median: 0
Mean: 55
Median: 50
Mean: 20
Median: 0
Work/household Minutes/week
Travel Minutes/week
Leisure Minutes/week
Work/household % contribution
Travel % contribution
Leisure % contribution

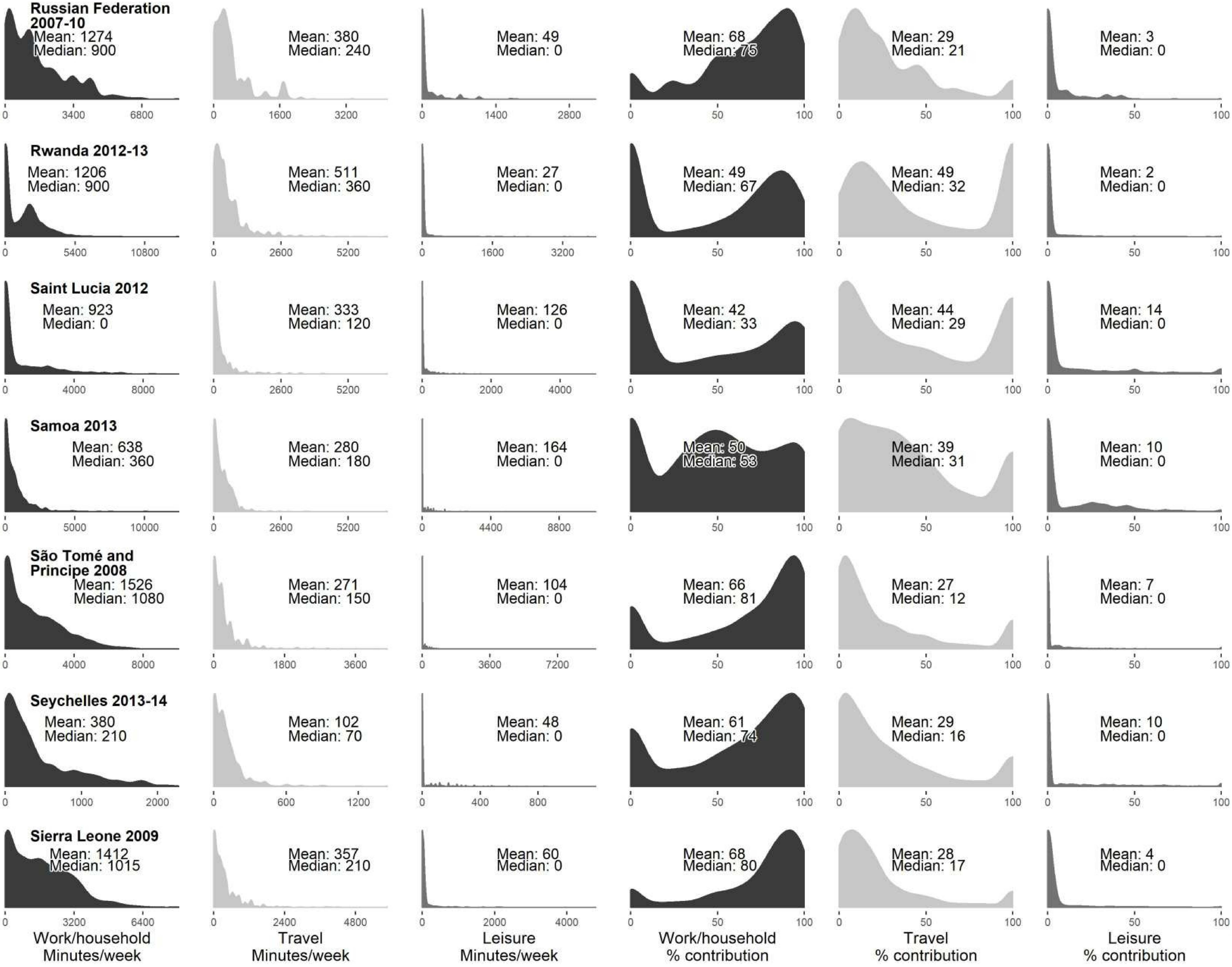
Russian Federation 2007-10
Mean: 1274
Median: 900
Mean: 380
Median: 240
Mean: 49
Median: 0
Mean: 68
Median: 75
Mean: 29
Median: 21
Mean: 3
Median: 0
Rwanda 2012-13
Mean: 1206
Median: 900
Mean: 511
Median: 360
Mean: 27
Median: 0
Mean: 49
Median: 67
Mean: 49
Median: 32
Mean: 2
Median: 0
Saint Lucia 2012
Mean: 923
Median: 0
Mean: 333
Median: 120
Mean: 126
Median: 0
Mean: 42
Median: 33
Mean: 44
Median: 29
Mean: 14
Median: 0
Samoa 2013
Mean: 638
Median: 360
Mean: 280
Median: 180
Mean: 164
Median: 0
Mean: 50
Median: 53
Mean: 39
Median: 31
Mean: 10
Median: 0
São Tomé and Principe 2008
Mean: 1526
Median: 1080
Mean: 271
Median: 150
Mean: 104
Median: 0
Mean: 66
Median: 81
Mean: 27
Median: 12
Mean: 7
Median: 0
Seychelles 2013-14
Mean: 380
Median: 210
Mean: 102
Median: 70
Mean: 48
Median: 0
Mean: 61
Median: 74
Mean: 29
Median: 16
Mean: 10
Median: 0
Sierra Leone 2009
Mean: 1412
Median: 1015
Mean: 357
Median: 210
Mean: 60
Median: 0
Mean: 68
Median: 80
Mean: 28
Median: 17
Mean: 4
Median: 0
Work/household Minutes/week
Travel Minutes/week
Leisure Minutes/week
Work/household % contribution
Travel % contribution
Leisure % contribution

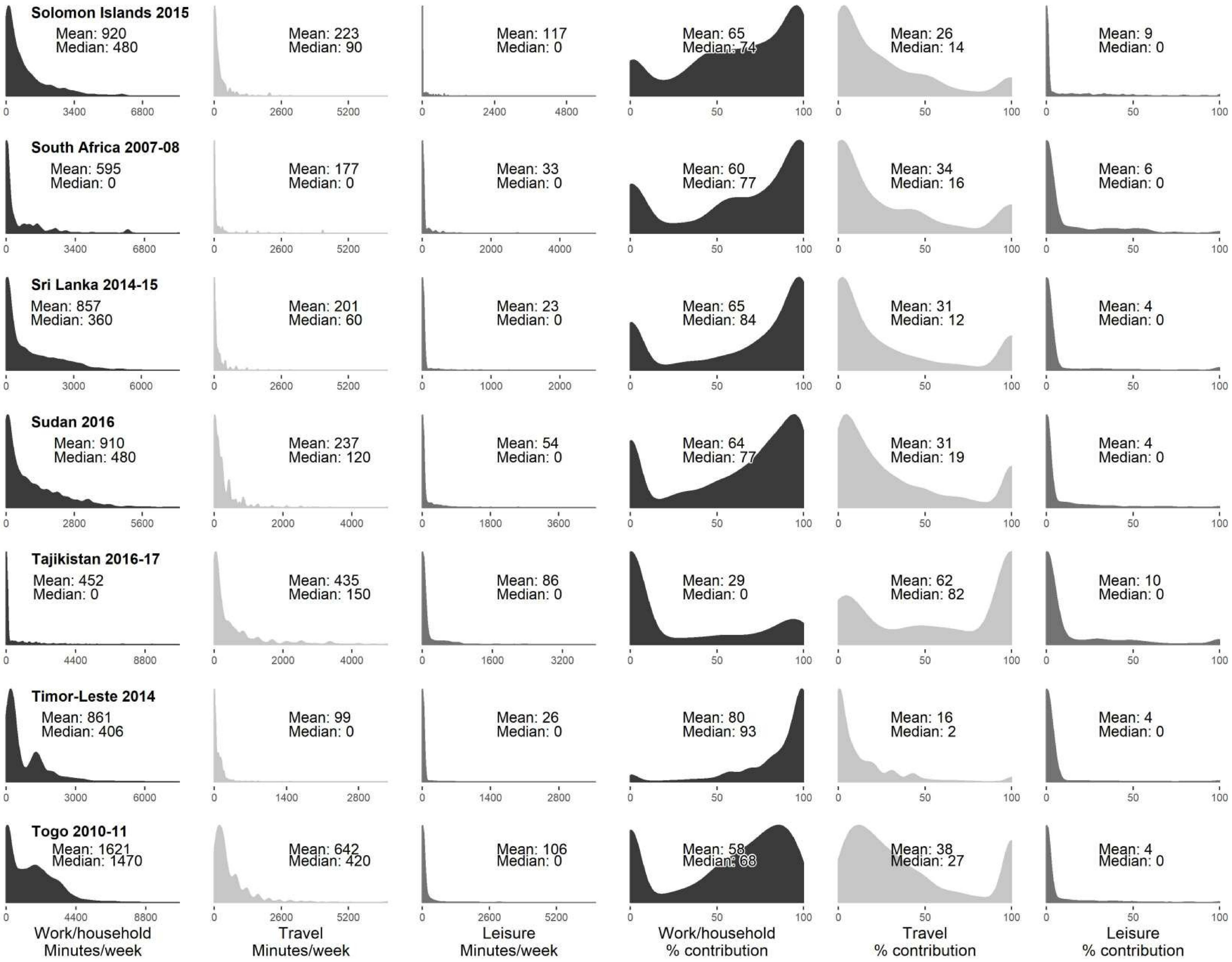
Solomon Islands 2015
Mean: 920
Median: 480
Mean: 223
Median: 90
Mean: 117
Median: 0
Mean: 65
Median: 74
Mean: 26
Median: 14
Mean: 9
Median: 0
South Africa 2007-08
Mean: 595
Median: 0
Mean: 177
Median: 0
Mean: 33
Median: 0
Mean: 60
Median: 77
Mean: 34
Median: 16
Mean: 6
Median: 0
Sri Lanka 2014-15
Mean: 857
Median: 360
Mean: 201
Median: 60
Mean: 23
Median: 0
Mean: 65
Median: 84
Mean: 31
Median: 12
Mean: 4
Median: 0
Sudan 2016
Mean: 910
Median: 480
Mean: 237
Median: 120
Mean: 54
Median: 0
Mean: 64
Median: 77
Mean: 31
Median: 19
Mean: 4
Median: 0
Tajikistan 2016-17
Mean: 452
Median: 0
Mean: 435
Median: 150
Mean: 86
Median: 0
Mean: 29
Median: 0
Mean: 62
Median: 82
Mean: 10
Median: 0
Timor-Leste 2014
Mean: 861
Median: 406
Mean: 99
Median: 0
Mean: 26
Median: 0
Mean: 80
Median: 93
Mean: 16
Median: 2
Mean: 4
Median: 0
Togo 2010-11
Mean: 1621
Median: 1470
Mean: 642
Median: 420
Mean: 106
Median: 0
Mean: 58
Median: 68
Mean: 38
Median: 27
Mean: 4
Median: 0
Work/household
Minutes/week
Travel
Minutes/week
Leisure
Minutes/week
Work/household
% contribution
Travel
% contribution
Leisure
% contribution

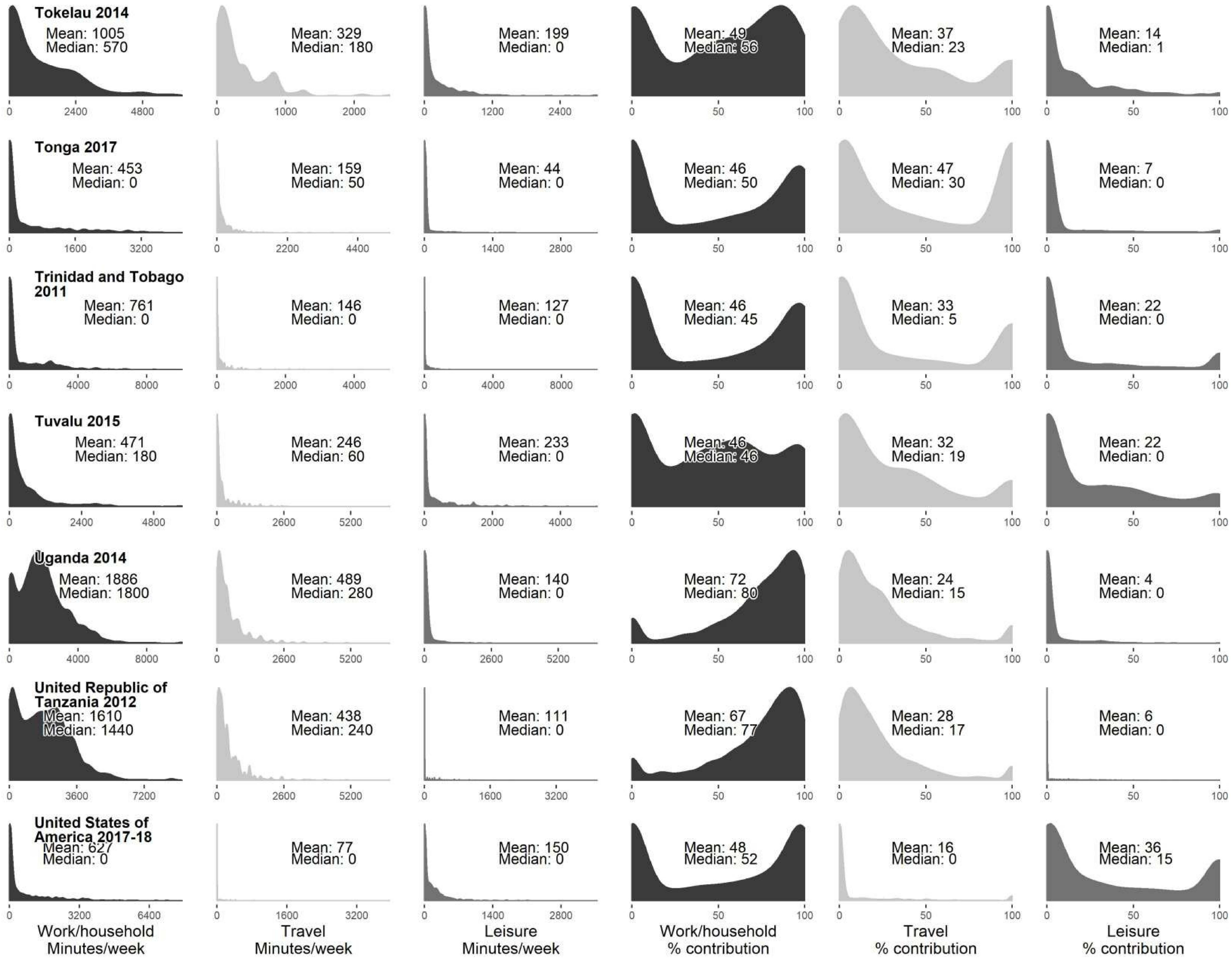
Tokelau 2014
Mean: 1005
Median: 570
Mean: 329
Median: 180
Mean: 199
Median: 0
Mean: 49
Median: 56
Mean: 37
Median: 23
Mean: 14
Median: 1
Tonga 2017
Mean: 453
Median: 0
Mean: 159
Median: 50
Mean: 44
Median: 0
Mean: 46
Median: 50
Mean: 47
Median: 30
Mean: 7
Median: 0
Trinidad and Tobago 2011
Mean: 761
Median: 0
Mean: 146
Median: 0
Mean: 127
Median: 0
Mean: 46
Median: 45
Mean: 33
Median: 5
Mean: 22
Median: 0
Tuvalu 2015
Mean: 471
Median: 180
Mean: 246
Median: 60
Mean: 233
Median: 0
Mean: 46
Median: 46
Mean: 32
Median: 19
Mean: 22
Median: 0
Uganda 2014
Mean: 1886
Median: 1800
Mean: 489
Median: 280
Mean: 140
Median: 0
Mean: 72
Median: 80
Mean: 24
Median: 15
Mean: 4
Median: 0
United Republic of Tanzania 2012
Mean: 1610
Median: 1440
Mean: 438
Median: 240
Mean: 111
Median: 0
Mean: 67
Median: 77
Mean: 28
Median: 17
Mean: 6
Median: 0
United States of America 2017-18
Mean: 627
Median: 0
Mean: 77
Median: 0
Mean: 150
Median: 0
Mean: 48
Median: 52
Mean: 16
Median: 0
Mean: 36
Median: 15
Work/household Minutes/week
Travel Minutes/week
Leisure Minutes/week
Work/household % contribution
Travel % contribution
Leisure % contribution

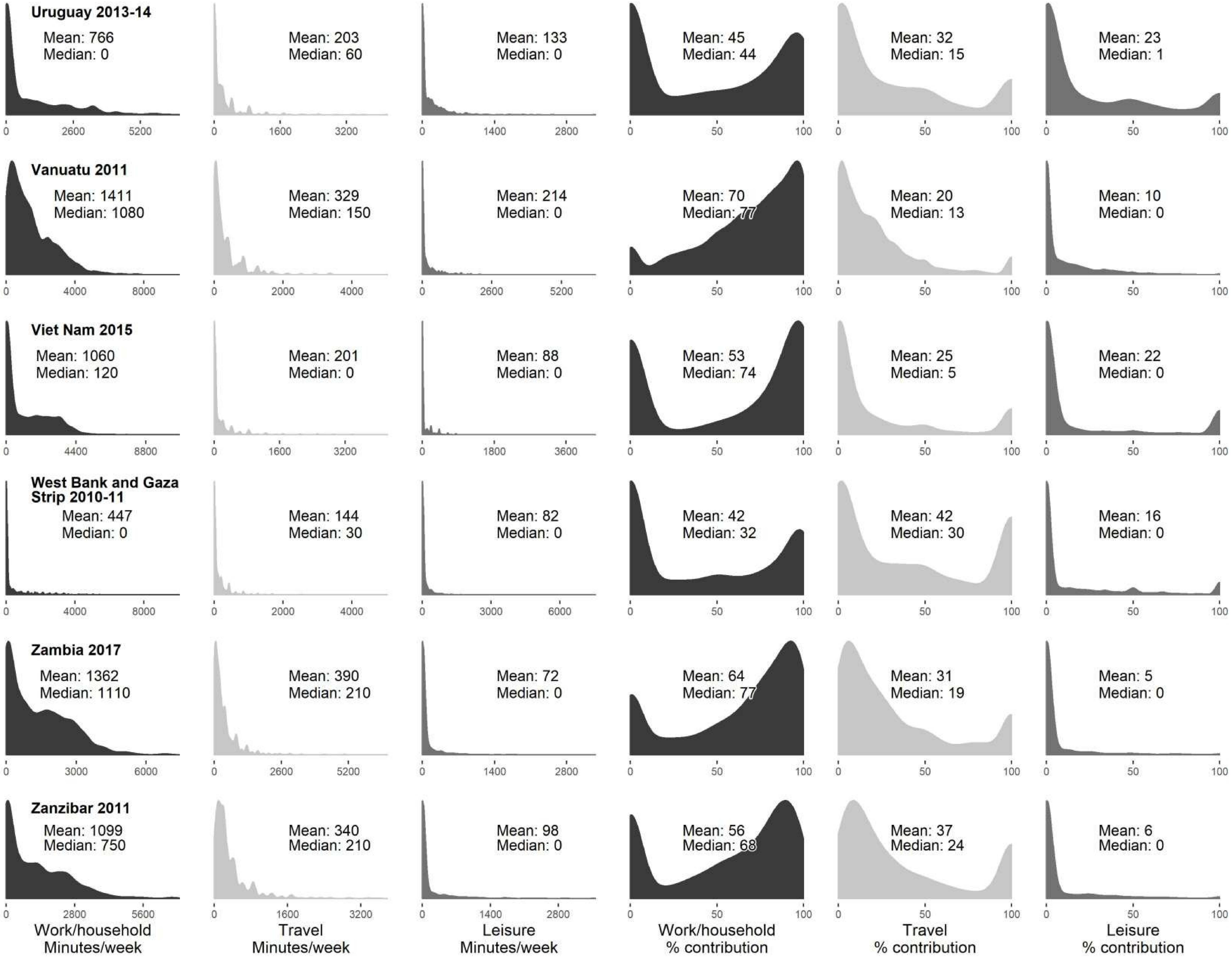
Uruguay 2013-14
Mean: 766
Median: 0
Mean: 203
Median: 60
Mean: 133
Median: 0
Mean: 45
Median: 44
Mean: 32
Median: 15
Mean: 23
Median: 1
Vanuatu 2011
Mean: 1411
Median: 1080
Mean: 329
Median: 150
Mean: 214
Median: 0
Mean: 70
Median: 77
Mean: 20
Median: 13
Mean: 10
Median: 0
Viet Nam 2015
Mean: 1060
Median: 120
Mean: 201
Median: 0
Mean: 88
Median: 0
Mean: 53
Median: 74
Mean: 25
Median: 5
Mean: 22
Median: 0
West Bank and Gaza Strip 2010-11
Mean: 447
Median: 0
Mean: 144
Median: 30
Mean: 82
Median: 0
Mean: 42
Median: 32
Mean: 42
Median: 30
Mean: 16
Median: 0
Zambia 2017
Mean: 1362
Median: 1110
Mean: 390
Median: 210
Mean: 72
Median: 0
Mean: 64
Median: 77
Mean: 31
Median: 19
Mean: 5
Median: 0
Zanzibar 2011
Mean: 1099
Median: 750
Mean: 340
Median: 210
Mean: 98
Median: 0
Mean: 56
Median: 68
Mean: 37
Median: 24
Mean: 6
Median: 0
Work/household Minutes/week
Travel Minutes/week
Leisure Minutes/week
Work/household % contribution
Travel % contribution
Leisure % contribution
